# Solvatochromism of new tetraphenylethene luminogens: integration of aggregation-induced emission and conjugation-induced rigidity for emitting strongly in both solid and solution state†

**DOI:** 10.1039/d4ra00719k

**Published:** 2024-02-16

**Authors:** Abdelreheem A. Saddik, Ahmed A. K. Mohammed, Satish K. Talloj, Adel M. Kamal El-Dean, Osama Younis

**Affiliations:** a Department of Chemistry, Faculty of Science, Assiut University Assiut 71516 Egypt abdelreheem@aun.edu.eg; b Intonation Research Laboratories Nacharam Hyderabad Telangana 500076 India; c Chemistry Department, Faculty of Science, New Valley University El-Kharga 72511 Egypt osamayounis@sci.nvu.edu.eg

## Abstract

In this study, we synthesized and characterized four tetraphenylethene (TPE) analogs, investigated their photophysical properties, and conducted quantum chemical calculations. Some molecules exhibited aggregation-induced emission enhancement behavior and showed efficient emission in both solid and solution states. Solvatochromism was observed in particular derivatives, with solvent polarity influencing either a bathochromic or hypsochromic shift, indicating the occurrence of photoinduced intramolecular charge transfer (ICT) processes. Quantum chemical calculations confirmed that variations in molecular packing and rigidity among the TPE analogs contributed to their diverse behavior. The study showcases aggregation in luminophores without significant impact on the excited state and highlights how minor alterations in terminal substituents can lead to unconventional behavior. These findings have implications for the development of luminescent materials. Furthermore, the synthesized compounds exhibited biocompatibility, suggesting their potential for cell imaging applications.

## Abstract 2

We synthesized and characterized TPE analogs, revealing AIE and solvatochromism. Quantum calculations confirmed diverse behavior due to molecular packing and rigidity. The compounds showed biocompatibility, suggesting potential for cell imaging.

## Graphical abstract

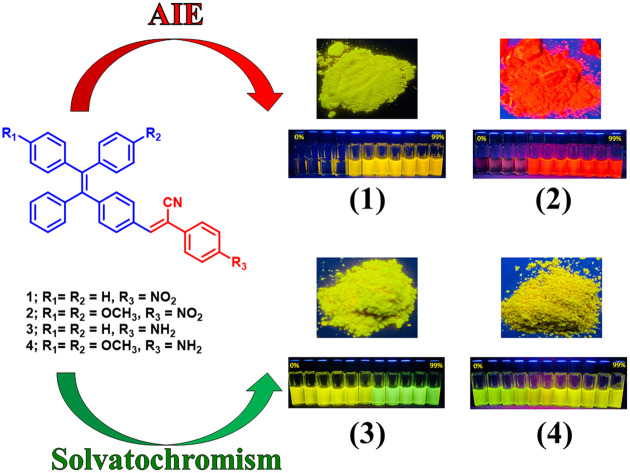

## 1. Introduction

The significance of highly efficient luminescent materials in the solid state cannot be overstated, as they have wide-ranging applications in materials science, optoelectronics, organic light-emitting devices (OLEDs), and bioprobes.^1–3^ Although it is essential to consider some classes of luminophores, such as cyanine derivatives that possess distinct structural characteristics,^4–6^ traditional organic luminophores often contain flat aromatic rings and exhibit efficient emission in diluted solutions. Still, their luminescence is weakened or completely quenched due to the formation of aggregates where the aggregate formation hinders the emission, which promotes exciton interactions and nonradiative pathways.^7^ This phenomenon is known as aggregation-caused quenching (ACQ)^8,9^ and restricts the use of luminogens in practical applications such as optoelectronic devices, imaging agents, and biosensors. Extensive efforts have been dedicated to mitigating or preventing the ACQ issue to achieve efficient emissions in the solid state. These strategies include cross-dipole packing,^10^ enhanced intramolecular charge transfer (ICT) transitions,^11^ the introduction of bulky substituents,^11–13^ and the formation of J-aggregates.^14^ Interestingly, unlike ACQ, Tang *et al.* discovered that a series of silole derivatives were non-emissive in diluted solutions but became highly luminescent when aggregated as solid films.^15^ They referred to this phenomenon as aggregation-induced emission (AIE), primarily attributed to the restriction of intramolecular motions in the solid state. The AIE effect offers a unique and powerful approach to constructing efficient solid emitters for diverse applications.^16–23^

Researchers have pursued two distinct approaches to address the challenge of achieving efficient solid-state emissions in luminescent dyes. On the one hand, they have aimed to impede the natural aggregation process in traditional rigid dyes that suffer from aggregation-caused quenching (ACQ). On the other hand, in the case of twisting dyes with aggregation-induced emission (AIE), their emission is remarkably efficient when aggregated. However, AIE luminophores are non-emissive when not aggregated. Furthermore, different sizes of aggregates may exhibit varying emissive behaviors.^24^ Moreover, aggregation-induced emission enhancement (AIEE) refers explicitly to the situation where the emission intensity of luminogens is further enhanced upon aggregation compared to their emission in the dispersed state.^25,26^ In other words, AIEE describes cases where the aggregation not only leads to a turn-on of fluorescence (AIE) but also causes an additional boost in emission intensity. AIEE materials exhibit even stronger emissions in the aggregated or solid-state environment than their individual molecular units, making them highly desirable for various applications.^27,28^ These disparities highlight a significant gap between ACQ and AIE compounds. Consequently, the question arises whether it is possible to combine the advantages of both ACQ and AIE dyes to create highly efficient luminogens that exhibit emission in both solution and solid states, thereby enabling broader applications.^29–31^ To achieve this, it is assumed that dual-state efficient luminogens should possess substantial rigidity with limited intramolecular motion in solution,^15^ while also adopting twisted conformations in the solid state to prevent detrimental exciton interactions. This requirement of substantial rigidity in solution combined with twisted conformations in the solid state within a single molecule may seem contradictory and unattainable. However, it has been discovered that triphenylamine exhibits strong fluorescence both in tetrahydrofuran (THF) solution and in the solid state with high photoluminescence efficiencies, bridging the gap between AIE and ACQ.^32,33^ Since its initial discovery by Tang and colleagues in 2007,^34^ tetraphenylethene (TPE) has gained widespread use in aggregation-induced emission (AIE) materials. This is primarily due to its propeller-like molecular structure, which effectively prevents the π–π accumulation of molecules, resulting in enhanced fluorescence brightness in aggregated or solid states.^35–43^ Consequently, TPE has become a highly regarded building block for AIE materials.^44^ Understanding the dynamic changes in the stereostructure of TPE is crucial for uncovering and comprehending its specific physical phenomena.^22,45^ Additionally, the propeller-like conformation of TPE resembles the characteristic photochromic behavior of diarylethenes.^7,46^ Solvatochromism refers to the changes in the absorption or emission spectrum of a molecule upon altering the polarity of the solvent.^47–50^ Molecules with low dipole moments in the ground state but significant dipole moments in the excited state, often due to ICT, exhibit a shift towards longer wavelengths (bathochromic shift) as solvent polarity increases.^49–51^ This shift occurs because more polar solvents provide excellent stabilization for the dipolar excited state.^52–54^ Recently, the polarity-dependent emission of substituted TPE derivatives was reported.^55^

This study presents the synthesis, photophysical analysis, and quantum chemical calculations of four TPE derivatives that exhibit desirable properties, including AIE, solvatochromism, and efficient emission in both the solid and solution states.

## 2. Results and discussion

### 2.1. Synthesis and characterization

**Scheme 1:**
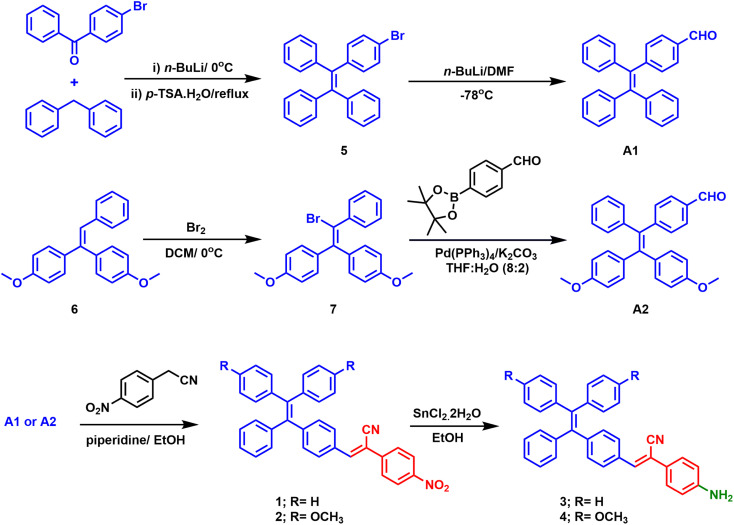
Synthesis routes to chromophores 1, 2, 3, and 4.

Compound A1 was synthesized using the procedure previously described in the literature.^56^ However, compound B2 was prepared according to the following procedures. In brief, the preparation of the Grignard solution of 4-methoxyphenyl bromide involved refluxing it with magnesium in the presence of the catalytic amount of iodine in THF for 3 hours. The resulting freshly prepared Grignard solution was then combined with a solution of methyl phenylacetate at 0 °C, and the reaction mixture was stirred for 24 hours at room temperature. Refluxing 1,1-bis(4-methoxyphenyl)-2-phenylethanol with toluene sulphonic acid (*p*-TsOH·H_2_O) yielded compound B. Bromination of compound B at room temperature resulted in the substitution of a bromo group (B1). Compound B2 was synthesized through a Suzuki–Miyaura cross-coupling reaction between compound 6 and 4-formylphenylboronic acid pinacol ester.^57,58^ Furthermore, the nitro-TPE derivatives (1 and 2) were obtained by performing Knoevenagel condensation of compounds A1 and B2 with *p*-nitrobenzyl cyanide in the presence of a catalytic amount of piperidine. Finally, the desired amino derivatives (3 and 4) were obtained by reducing the nitro group using stannous chloride dihydrate (SnCl_2_·2H_2_O) in ethanol (Scheme 1).^59^ The structures of the newly synthesized compounds were confirmed using ^1^H-NMR, ^13^C-NMR spectroscopy, and high-resolution mass spectrometry. Derivative 3 has been previously used as a polymer building block.^60^

### 2.2. Photophysical properties

**Fig. 1:**
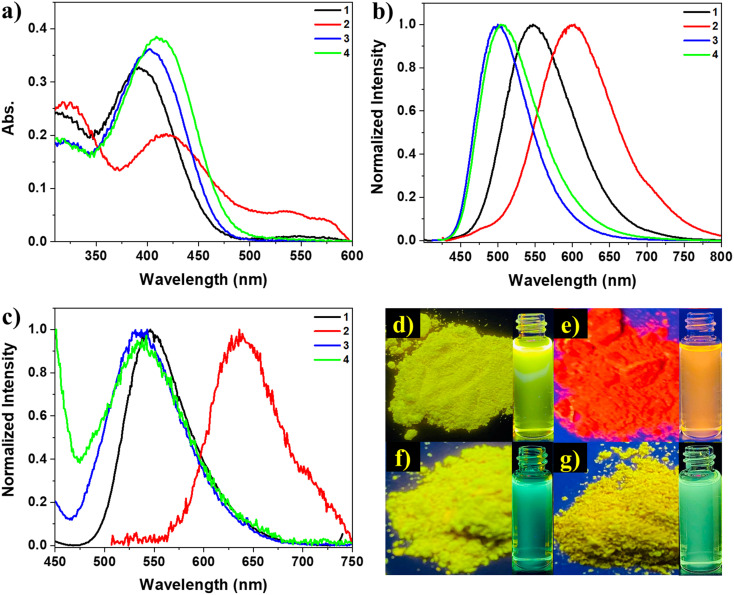
(a) Absorption spectra of solutions (5 × 10^−5^ mol L^−1^). (b) Normalized emission spectra of solutions (*λ*_ex_ = 391, 420, 400, and 409 nm for 1, 2, 3, and 4, respectively, 5 × 10^−5^ mol L^−1^). (c) Normalized emission spectra of solids (*λ*_ex_ = 391, 420, 400, and 409 nm for 1, 2, 3, and 4 respectively). (d–g) Photos taken under UV irradiation for solids of compounds 1, 2, 3, and 4, respectively {insets: optical images of the solutions in dioxane}.

The optical properties of the materials under investigation were thoroughly examined. In the DMSO solution, the absorption spectra of these compounds displayed π–π* transition bands at 391, 420, 400, and 409 nm for chromophores 1, 2, 3, and 4, respectively, along with another band at about 320 nm, as shown in Fig. 1a. The absorption spectra indicated that these materials had similar electronic ground states in dilute solutions, as there was no aggregation of individual molecules due to the solvation effect.^61^ However, the amino-substituted derivatives (3 and 4) exhibited a more intense absorption peak at longer wavelengths, suggesting that the substituted functional group (nitro or amino) influenced the conjugation of the molecules. The peaks of the amino-substituted derivatives had strong molar extinction coefficients, likely corresponding to intramolecular charge transfer absorptions involving the amino and cyano groups.^62^ The molar extinction coefficient (*ε*) at the maximum absorption wavelength for these compounds was approximately 4 × 10^3^ L mol^−1^ cm^−1^. The photoluminescence behavior of compounds 1–4 was investigated in both powder and solution forms, using dioxane as a solvent, where the dioxane solutions of the four compounds exhibited strong emission under UV illumination, as depicted in Fig. 1d–g. The emission spectra of the four solutions displayed similar shapes, indicating that the compounds shared the same luminescent core. Similarly, the similar spectra of the powders (Fig. 1c) suggested that the aggregation of these molecules did not significantly affect the excited state. Compounds 1, 2, 3, and 4 exhibited emission spectra with *λ*_max_ = 546, 600, 497, and 505 nm in their solutions and 546, 637, 533, and 538 nm in their powders, respectively. Therefore, it could be observed with the naked eye that compounds 1, 3, and 4 emitted yellow to green light under UV irradiation, while compound 2 emitted red light Fig. 1d–g. The red-shifted emission of compound 2 compared to the others may be attributed to the extended conjugation resulting from the electron-donating and electron-withdrawing terminal groups (methoxy and nitro). The luminescence spectra of compounds 2, 3, and 4 in the powder form exhibited a red shift with *λ*_max_ = 637, 533, and 538 nm, respectively, compared to their solution spectra with *λ*_max_ = 600, 497, and 505 nm. This red shift in the emission is commonly observed in organic components as the molecules aggregate in powders and concentrated solutions, leading to strengthened π–π interactions between neighboring molecules.^63–65^ However, compound 1 did not display this red shift upon aggregation, maintaining a *λ*_max_ of 546 nm in both solid and solution emissions. The absence of the red shift suggests that π–π interactions are not favored in the powder aggregates of compound 1 compared to its solution. Additionally, intermolecular interactions within its aggregates may also hinder the elongation of the conjugation and prevent the red shift in powder emission. Conversely, the conjugation-induced rigidity (CIR) may increase planarity and prevent a blue shift in the solution.^31,32^ Consequently, density functional theory (DFT) calculations were performed to confirm that the four derivatives exhibited different planarity, intermolecular interactions, and small intermolecular distances within dimers, potentially influencing their photophysical behavior.

**Fig. 2:**
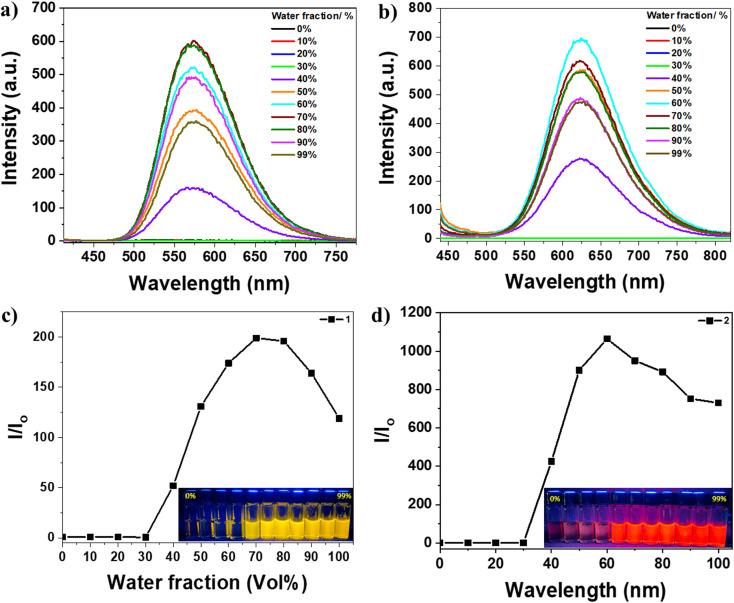
Emission spectra at *λ*_ex_ = 391 and 420 nm for 1 and 2, respectively; (a) 1 and (b) 2 solutions (5 × 10^−5^ mol L^−1^) in DMSO with various H_2_O fractions. Variation of emission intensity of (c) 1 and (d) 2 {insets: optical images of the solutions with varied water content under UV light (365 nm)}.

**Fig. 3:**
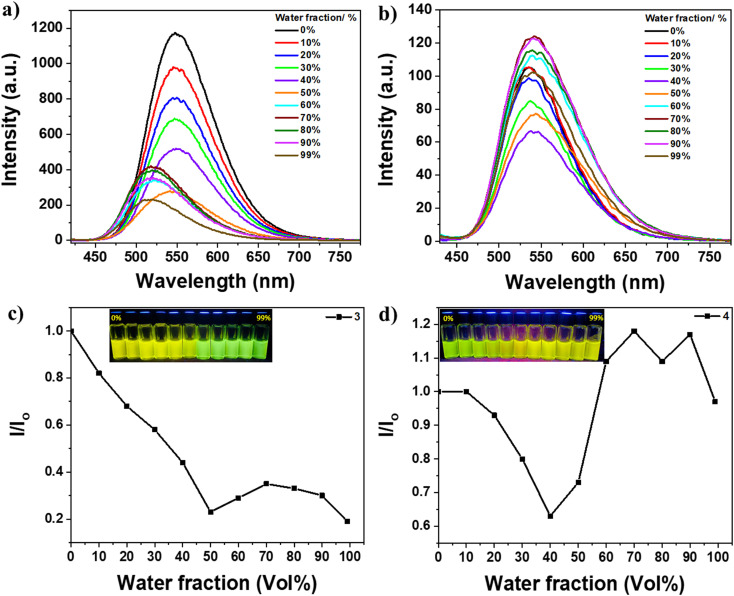
Emission spectra at *λ*_ex_ = 400 and 409 nm for 3 and 4, respectively; (a) 3 and (b) 4 solutions (5 × 10^−5^ mol L^−1^) in DMSO with various H_2_O fractions. Variation of emission intensity of (c) 3 and (d) 4 {insets: optical images of the solutions with varied water content under UV light (365 nm)}.

To confirm the AIEE behavior of the compounds under investigation, their emission spectra were measured for different mixtures of DMSO and water, which act as good and poor solvents, respectively (Fig. 2 and 3). The TPE dyes studied were soluble in common organic solvents such as DMSO, acetonitrile, chloroform, and THF but insoluble in water. DMSO was chosen as the solvent for this experiment because the dilute DMSO solutions of these dyes exhibited negligible luminescence. However, the addition of water, a non-solvent for the TPE derivatives, significantly impacted their photoluminescence processes. In molecularly dissolved solutions with low water fractions, the isolated species of compounds 1 or 2 displayed a photoluminescence spectrum that appeared as a nearly flat line parallel to the *x*-axis (Fig. 2a and b). However, upon adding a large amount of water, the mixture became visibly emissive (as seen in the inset photos under UV illumination). Since water is a nonsolvent for these TPE-based derivatives, their molecules must aggregate in the aqueous mixture solution with high water fractions. The aggregates of compounds 1 and 2 in the aqueous mixture exhibited significantly enhanced fluorescence compared to their behavior in DMSO, with an increase in photoluminescence intensity of 200-fold and 1000-fold, respectively. It is evident that aggregates' formation induces these compounds' emissions. Interestingly, the maximum photoluminescence intensity of compounds 1 and 2 was observed at 70% and 60% water fractions, respectively, rather than at the highest water fraction in the mixture solution. This observation may be attributed to a change in the packing order of aggregates from a crystalline state to an amorphous state,^65^ or it could be influenced by the solubility of compounds 1 and 2 in DMSO/water mixtures with high water fractions.^66^ The emission of compounds 1 and 2 is induced by the formation of aggregates, indicating that they exhibit AIE activity (Fig. 2c and d).

In contrast, compounds 3 and 4 did not exhibit typical AIE behavior, as there was no enhancement in emission intensity upon the addition of higher water fractions (Fig. 3). The photos under UV light in Fig. 3c and d insets clearly show that dyes 3 and 4 emit strongly in all solvent fractions. Additionally, these two materials exhibit emission in the solid state as well (Fig. 1d). Therefore, it can be concluded that dyes 3 and 4 possess emissive properties both in the solution and aggregate states, bridging the gap between AIE and ACQ. In the solution with low water fractions, the emission arises from conjugation-induced rigidity (CIR), which prevents intramolecular rotation.^32^ Conversely, in the aggregated states with higher water fractions, the emission originates from AIE resulting from aggregate formation and restriction of intramolecular rotation (RIR). The trend of the increase or decrease in emission intensity can be attributed to the combined effect of both CIR and RIR, which may either work in tandem or oppose each other. For instance, the formation of aggregates diminishes the CIR effect due to the creation of short contacts that induce distortion of molecular planarity, reducing the conjugation length and weakening the rigidity, thereby allowing molecular motion. This phenomenon can also account for the observed blue shift in the emission of dye 3 at high water fractions. It can be speculated that CIR leads to high planarity of the molecules in solutions with low water fractions, enhancing conjugation and increasing the emission wavelength. On the other hand, at high water fractions, the formation of short contacts distorts molecular planarity and reduces the conjugation length, resulting in the observed blue shift.

**Fig. 4:**
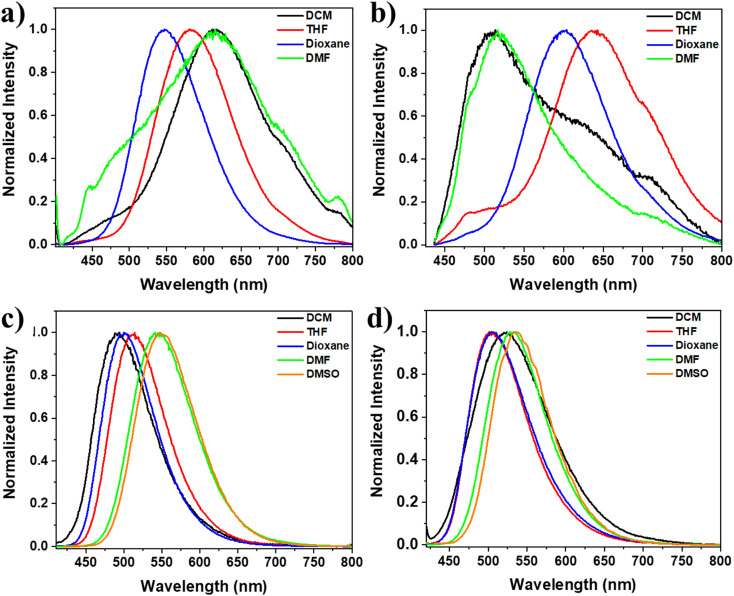
Normalized emission spectra at *λ*_ex_ = 391, 420, 400, and 409 nm for 1, 2, 3, and 4, respectively; (a) 1, (b) 2, (c) 3, and (d) 4 in solvents of different polarity (5 × 10^−5^ mol L^−1^).

Furthermore, we also examined the solvatochromic characteristics of the TPE-based compounds in various solvents (Fig. 4). The luminescent properties of solutions containing all four dyes exhibited striking changes when the solvent was altered. This phenomenon is commonly observed in molecules with a donor–acceptor (D–A) structure, wherein photoinduced ICT takes place from the electron-donating substituent group to the electron-accepting group of the chromophore in the singlet excited state.^67,68^ For instance, dye 3 displayed a remarkable change in fluorescence color depending on the polarity of the solvent: blue in dichloromethane, green in THF, and yellow in DMF and DMSO. A significant red shift was observed in the emission spectrum, with the wavelength changing from 491 to 546 nm as the solvent polarity increased from less polar (dichloromethane) to more polar (DMSO) solvent. This suggests that the HOMO–LUMO gap becomes narrower in polar solvents, indicating the occurrence of photoinduced ICT from the electron-donating substituent group to the electron-accepting group of the chromophore in the singlet excited state. Similarly, dye 4 exhibited a red shift in the emission spectrum, with the wavelength changing from 503 nm in less polar solvents like THF and dioxane (greenish-blue emission) to 531 and 535 nm in more polar solvents like DMF and DMSO (yellow emission), Fig. 4 and S1.†

On the other hand, increasing the solvent polarity of solutions containing dyes 1 and 2 resulted in a bathochromic shift with some solvents and a hypsochromic change with others. For example, increasing the solvent polarity of the dye 1 solution, including dichloromethane, THF, dioxane, and DMF, led to changes in the *λ*_max_ values of 615, 582, 546, and 616, respectively. Furthermore, dye 2 solution, including dichloromethane, THF, dioxane, and DMF, led to changes in the *λ*_max_ values of 509, 637, 600, and 517, respectively (Fig. 4 and S1†). This behavior can be attributed to the varying solvation of the excited state in different solvents, regardless of their polarity order, and it can also be due to solvent-induced changes in the molecular geometry. In other words, the π-system of the molecules may become more or less planar due to changes in the surrounding solvent.^69^ The discrepancy in the solvatochromic behavior between derivatives 1 and 2, which contain the nitro group, and derivatives 3 and 4, which have the amino group, can be attributed to the significant influence of these groups on the molecular geometry, molecular packing, intermolecular interactions, and solute–solvent interactions. The role of these groups was confirmed by the DFT calculations, which are explained in more detail below.

### 2.3. Quantum-chemical calculations

**Fig. 5:**
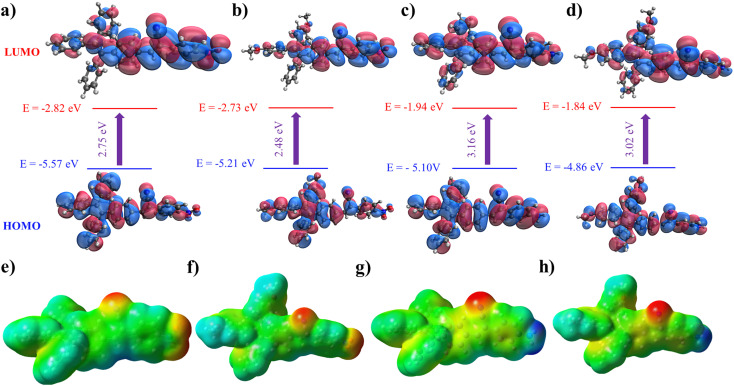
The HOMO and LUMO of (a) 1, (b) 2, (c) 3, and (d) 4, along with their energies calculated at the B3LYP-D3BJ/6-31G(d) level. The molecular electrostatic potential of (e) 1, (f) 2, (g) 3, and (h) 4 at the B3LYP-D3BJ/6-31G(d) level. Color: red is the most negative (nucleophilic atoms), and blue is the most negative (electrophilic atoms).

Crystal structures play a crucial role in understanding the molecular arrangement and intermolecular interactions within compounds. However, despite our diligent efforts, we encountered challenges in obtaining suitable single crystals for X-ray diffraction analysis to determine the crystal structures of the new compounds. So, we employed DFT calculations as an alternative approach to compensate for the unavailability of experimental crystal structures. DFT calculations are widely accepted and utilized without crystallographic data to gain insights into compounds' electronic structure and other properties. Extensive DFT calculations were performed to study the compounds' electronic structure, molecular properties, and other relevant characteristics, providing valuable insights into their behavior and potential applications. We conducted DFT calculations to gain insights into the three-dimensional structure and properties of the four molecules. Specifically, we utilized the B3LYP-D3BJ/6-31G(d) level of theory to calculate the HOMO (highest occupied molecular orbital) and LUMO (lowest unoccupied molecular orbital) energies, as well as their distributions, for each molecule (refer to Fig. 5a–d). These calculations allowed us to examine the electron distribution within these molecules in detail. The results indicate that both the HOMO and LUMO are extensively delocalized throughout the conjugated system for all four molecules, suggesting a high degree of conjugation. Furthermore, we determined the calculated band gaps for molecules 1, 2, 3, and 4, which were found to be 2.75, 2.48, 3.16, and 3.02 eV, respectively. In Fig. 5e–h, we present the electrostatic potential for the four molecules. In molecules 3 and 4, the negative potential is concentrated around the nitrogen atom of the cyano group, while the most positively charged atoms are the hydrogens of the amino group. In contrast, for molecules 1 and 2, the cyano and nitro groups exhibit the highest electron density within the molecule.

**Fig. 6:**
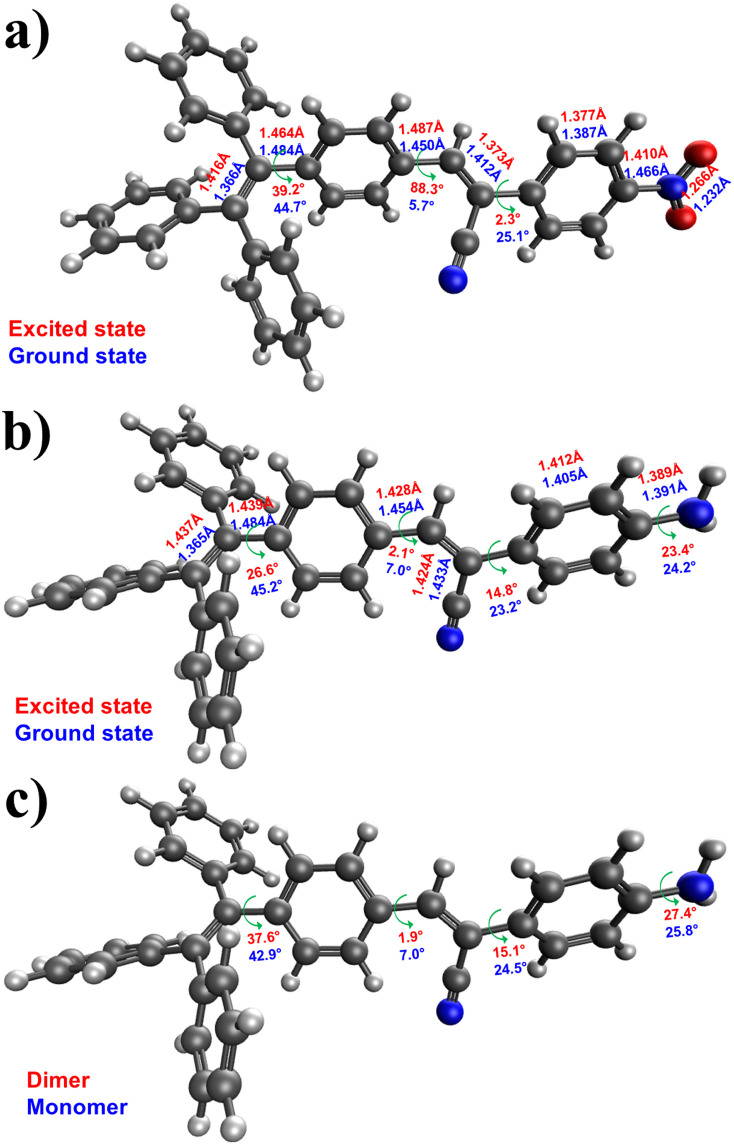
Critical changes in structural parameters between the ground and excited states of (a) 1 and (b) 3 were calculated at the B3LYP-D3BJ/6-31G(d) level. Color: grey = C; blue = N; red = O. (c) Key changes in structural parameters between the monomer and dimer of molecule 3 calculated at the B3LYP-D3BJ/6-31G(d) level.

To accurately illustrate the AIE characteristic of the dyes, we conducted calculations to determine the structural variations between the excited and ground states. The structure of the first excited state was optimized using the same level of theory. Fig. 6a and b comprehensively compares the structural parameters between the ground and excited states of molecules 1 and 3. In general, the excited states of both molecules exhibit a higher degree of planarity than the ground state. Fig. 6c illustrates the significant changes in structural parameters for both the monomer and dimer of molecule 3. Overall, we observe subtle modifications in both molecules to optimize the interaction between the rings of the two molecules, thereby promoting stronger intermolecular interactions. The capability of these molecules to assemble is crucial for their photoluminescence. DFT calculations demonstrate the flexible nature of these molecules, allowing for free rotation around single bonds. For instance, the rotation of the aniline and TPE moieties possesses a relatively low energy barrier of 4.2 and 5.5 kcal mol^−1^, respectively. These rotations are feasible at room temperature. However, as the molecules aggregate, such intramolecular rotations are hindered, enhancing emission intensity.

**Fig. 7:**
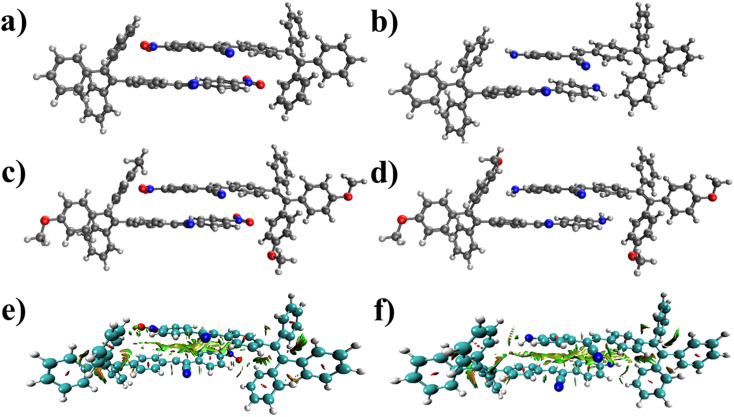
The geometry of the most stable dimers of molecules (a) 1, (b) 2, (c) 3, and (d) 4 computed at the B3LYP-D3BJ/6-31G(d) level. The intermolecular interactions in the dimers of molecules (e) 1 and (f) 3 obtained by the Multiwfn program^70^ on geometries optimized at the B3LYP-D3BJ/6-31G(d) level. Color code: blue indicates hydrogen bonding (absent here), red indicates repulsion (steric effect), and green indicates van der Waals interactions.

Fig. 7a–d presents the optimized structures of the four molecules' dimers. Various binding modes were examined, and the most stable dimer structures were selected. π–π stacking interactions primarily characterize the structures depicted in Fig. 7. The dimers of molecules 1, 2, 3, and 4 exhibit 27.7, 28.0, 29.4, and 29.7 kcal mol^−1^ binding energies, respectively. These substantial binding energies indicate the stability of the four dimers. The average intermolecular distance within these dimers is less than 3.35 Å, which restricts the rotation of the rings of the four molecules around single bonds. Additionally, Fig. 7e and f illustrate the reduced density gradient (RDG) plots for the dimers of molecules 1 and 3. These plots demonstrate that van der Waals forces primarily govern the interactions between the two molecules in both dimers.

### 2.4. Cytotoxicity

We further evaluated the cytotoxicity of synthesized compounds 1, 2, 3, and 4 towards two different cancer cell lines: (i) the human hepatoma cancer cells (HepG2) and (ii) the permanent cell line established from male hepatoma tissue (hereafter Huh-7) for 24 h. The tested compounds were dissolved in DMSO and then serially diluted in a complete culture medium. According to the cell viability results shown in Fig. S10,† HepG2 cells exposed to 1, and 2 show a cell survival ratio of around 71%, and 76% respectively. Whereas 3 and 4 show cell survival ratios around 87%, and 75% respectively at the concentration of 124 μM after 24 h of treatment. On the other hand, the MTT assay results for Huh-7 cells showed that compared with those in the control group, the cell viability was around 93%, 86%, 91%, and 98% in 1, 2, 3, and 4 at the concentration of 124 μM after 24 h of treatment, respectively. These results indicate the biocompatibility of the synthesized compounds which may be useful for cell imaging applications (Fig. S2 and S3†).^59^

## 3. Conclusion

In conclusion, this study presents the synthesis and characterization of a set of four TPE analogs. The investigation reveals their diverse properties, including AIEE behavior, efficient emission in both solid and solution states, and solvatochromism. Quantum chemical calculations supported these findings, highlighting the impact of molecular packing and rigidity on the observed behavior. Importantly, this study demonstrates that minor alterations in terminal substituents can lead to unconventional behavior in TPE luminophores. These findings not only contribute to our understanding of aggregation in luminophores but also offer new possibilities for the development of luminescent materials. Additionally, the synthesized compounds exhibited solvent-dependent fluorescence color changes, indicating the occurrence of photoinduced ICT processes. Furthermore, the cytotoxicity evaluation demonstrated the biocompatibility of the synthesized compounds, suggesting their potential suitability for cell imaging applications. This comprehensive investigation opens up avenues for further research and applications in the field of luminescent materials.

## 4. Experimental

### 4.1. Materials and methods

4-Bromobenzophenone, 4,4′-dimethoxybenzophenone, and diphenylmethane were purchased from Alfa Aesar. 4-Formylphenylboronic acid pinacol ester and *p*-nitrobenzyl cyanide were purchased from ThermoFisher Scientific. Anhydrous grade solvents were purchased from Sigma-Aldrich and Acros Organics. The purity of the compounds was confirmed using ^1^H NMR, ^13^C NMR, and MS techniques. NMR spectra of the tetraphenylethene compounds were recorded at VARIAN AS500 MHz in deuterated solvents. Coupling constants (*J*) are denoted in Hz and chemical shifts (*δ*) in ppm. Multiplicities are denoted as follows: s = singlet, d = doublet, t = triplet, m = multiplet, br = broad. High-resolution mass spectra (HRMS) were recorded on a Micromass Q-TOF MS spectrometer.

### 4.2. Synthesis and characterization

#### General procedure for Knoevenagel condensation to prepare compounds 1 and 2 (ref. 59)

The proper aldehydes (5 mmol), ethanol (150 mL), *p*-nitrobenzylcyanide (0.81 g, 5 mmol), and piperidine (0.25 mL) were added into a one-neck flask. The mixture was refluxed at 80 °C for 4 h. After cooling to room temperature, the crystal that had formed was filtered off and washed with ethanol three times.

##### (*Z*)-2-(4-Nitrophenyl)-3-(4-(1,2,2-triphenylvinyl)phenyl)acrylonitrile (1)

As a yellow solid material in 79% yield. ^1^H NMR (500 MHz, CDCl_3_, 25 °C): *δ* = 7.00–7.05 (m, 6H; 6CH), 7.10–7.20 (m, 11H; 11CH), 7.55 (s, 1H; CH), 7.71 (d, *J* = 10 Hz, 2H; 2CH), 7.80 (d, *J* = 10 Hz, 2H; 2CH), 8.29 (d, *J* = 10 Hz, 2H; 2CH); ^13^C NMR (125 MHz, CDCl_3_, 25 °C): *δ* = 108.99, 117.98, 125.01, 127.23, 127.50, 127.69, 128.37, 128.59, 129.95, 131.90, 131.98, 132.79, 140.44, 141.51, 143.81, 145.77, 148.42, 148.54; HRMS [ESI^+^]: *m*/*z* (%): calcd 505.1911, obsvd 505.1907 [M + H]^+^.

##### (*Z*)-3-(4-(2,2-Bis(4-methoxyphenyl)-1-phenylvinyl)phenyl)-2-(4-nitrophenyl)acrylonitrile (2)

As a red solid material in 79% yield. ^1^H NMR (500 MHz, CDCl_3_, 25 °C): *δ* = 3.74 (s, 3H; CH_3_), 3.76 (s, 3H; CH_3_), 6.66 (dd, *J* = 7.5, 17.5 Hz, 4H; 4CH), 6.96 (dd, *J* = 7.5, 12.5 Hz, 4H; 4CH), 7.15 (dd, *J* = 5, 10 Hz, 2H; 2CH), 7.55 (s, 1H; CH), 7.72 (d, *J* = 5 Hz, 2H; 2CH), 7.81 (d, *J* = 5 Hz, 2H; 2CH), 8.29 (d, *J* = 5 Hz, 2H; 2CH); ^13^C NMR (125 MHz, CDCl_3_, 25 °C): *δ* = 55.08, 55.12, 107.92, 113.03, 113.29, 117.40, 124.32, 126.50, 127.94, 129.34, 130.38, 131.39, 132.16, 132.66, 135.72, 137.98, 140.92, 142.20, 143.56, 145.17, 148.62, 158.37, 158.56; HRMS [ESI^+^]: *m*/*z* (%): calcd 565.2122, obsvd 565.2116 [M + H]^+^.

#### General procedure for reduction of nitro compounds^59^

A flask equipped with a magnetic stirrer received a solution of 150 mL of ethanol and 2 mmol of a nitro compound. Subsequently, SnCl_2_·2H_2_O (11 mmol) was added, and the mixture was refluxed for 0.5 hours. The resulting mixture was neutralized to weak basicity by adding saturated NaHCO_3_, and then diluted with approximately 50 mL of water. The diluted mixture was subjected to extraction using dichloromethane. The combined organic phase was dried using MgSO_4_, filtered, and concentrated, resulting in the formation of a deep orange solid compound. The crude material obtained was further purified by performing silica gel column chromatography, using a DCM to DCM/MeOH (9.7 : 0.3) as an eluent.

##### (*Z*)-2-(4-Aminophenyl)-3-(4-(1,2,2-triphenylvinyl)phenyl)acrylonitrile (3)

As a yellow solid material in 70% yield. ^1^H NMR (500 MHz, CDCl_3_, 25 °C): *δ* = 6.69 (d, *J* = 10 Hz, 2H; 2CH), 7.05–7.15 (m, 15H; 15CH), 7.24 (s, 1H; CH), 7.44 (d, *J* = 10 Hz, 2H; 2CH), 7.62 (d, *J* = 5 Hz, 2H; 2CH); ^13^C NMR (125 MHz, CDCl_3_, 25 °C): *δ* = 110.64, 115.08, 118.45, 126.65, 127.13, 127.66, 127.80, 127.87, 128.35, 131.29, 131.30, 131.36, 131.79, 138.11, 140.15, 142.03, 143.27, 143.38, 143.44, 145.60, 147.43; HRMS [ESI^+^]: *m*/*z* (%): calcd 475.2169, obsvd 475.2178 [M + H]^+^.

##### (*Z*)-2-(4-Aminophenyl)-3-(4-(2,2-bis(4-methoxyphenyl)-1-phenylvinyl)phenyl)acrylonitrile (4)

As a yellow solid material in 83% yield. ^1^H NMR (500 MHz, CDCl_3_, 25 °C): *δ* = 3.73 (s, 3H; CH_3_), 3.75 (s, 3H; CH_3_), 6.60–6.70 (m, 6H; 6CH), 6.90–7.00 (m, 4H; 4CH), 7.04 (dd, *J* = 5, 10 Hz, 2H; 2CH), 7.08 (d, *J* = 5 Hz, 2H; 2CH), 7.10–7.15 (m, 2H; 2CH), 7.23 (s, 1H; CH), 7.43 (d, *J* = 5 Hz, 2H; 2CH), 7.60 (d, *J* = 10 Hz, 2H; 2CH); ^13^C NMR (125 MHz, CDCl_3_, 25 °C): *δ* = 55.08, 55.11, 110.33, 113.00, 113.23, 115.07, 118.52, 124.79, 126.31, 127.10, 127.83, 128.40, 131.42, 131.78, 131.83, 132.60, 132.62, 135.98, 136.05, 138.24, 138.39, 141.27, 143.84, 146.30, 147.37, 158.20, 158.37; HRMS [ESI^+^]: *m*/*z* (%): calcd 535.2380, obsvd 535.2390 [M + H]^+^.

### 4.3. Photophysical properties

The UV-Vis absorption spectra were acquired using a HP8453 UV-visible spectrophotometer. For the measurement of steady-state photoluminescence spectra, a Hitachi F-4500 fluorescence spectrometer.

### 4.4. Quantum-chemical calculations

The optimization of the ground state geometry for the four molecules was carried out using DFT with the B3LYP functional and the D3BJ correction, along with the 6-31G(d) basis set.^71,72^ The inclusion of the D3BJ correction is crucial to account for long-range and noncovalent interactions, such as hydrogen bonding and π–π stacking, which play a significant role in this study and are essential for obtaining accurate intermolecular interactions.^73^ Multiple conformers were considered during the optimization process, and the one with the lowest energy was selected as the global minimum. This selection was further confirmed by examining the harmonic vibrational frequency. The binding energies (Δ*E*_b_) for the dimers were subsequently calculated at the same level of theory using the equation Δ*E*_b_ = *E*_dimer_ − 2*E*_monomer_.

### 4.5. Cytotoxicity assays

To determine the cytotoxic effect of all the compounds over a 24 h period, the 5-dimethylthiazol-2-yl-2,5-diphenyl-tetrazolium bromide (MTT) assay was performed. HepG2 and Huh-7 cells were trypsinized and plated to ∼70% confluence in 96-well plates 24 h before treatment. Before the treatment of compounds, the DMEM was removed and replaced with fresh DMEM the treated cells with different concentrations form the synthesized compounds were incubated for 24 h at 37 °C and under 5% CO_2_. Subsequently, the cells were treated with 0.5 mg mL^−1^ MTT and incubated for an additional 4 h (37 °C, 5% CO_2_). Then, DMEM was removed, the formazan crystals were dissolved in DMSO (150 mL per well), and the absorbance was recorded. The reported percent cell survival values are relative to untreated control cells.

## Conflicts of interest

There are no conflicts to declare.

## Supplementary Material

RA-014-D4RA00719K-s001
