# Supplementary material for: Solvatochromism of new tetraphenylethene luminogens: integration of aggregation-induced emission and conjugation-induced rigidity for emitting strongly in both solid and solution state
Source: RSC Adv. 2024 Feb 16;14(9):6072–84. doi: 10.1039/d4ra00719k (PMC10870197; doi:10.1039/d4ra00719k)
Supplement: RA-014-D4RA00719K-s001 [file RA-014-D4RA00719K-s001.pdf]

Electronic Supplementary Material (ESI) for RSC Advances.
This journal is © The Royal Society of Chemistry 2024

Supporting Information

Solvatochromism of New Tetraphenylethene Luminogens: Integration of Aggregation-Induced Emission and Conjugation-Induced Regidity for Emitting Strongly in Both Solid and Solution

Abdelreheem Abdelfatah Saddik,[a#]* *Ahmed A. K. Mohammed,*[a#] *Satish Kumar Talloj,*[b] *Adel M. Kamal El-dean,*[a] *Osama Younis*[c]*

[a]Department of Chemistry, Faculty of Science, Assiut University, Assiut, 71516, Egypt.

[b]Intonation Research Laboratories, Nacharam, Hyderabad, Telangana 500076, India.

[c]Chemistry Department, Faculty of Science, New Valley University, El-Kharga, 72511, Egypt.

[#] Abdelreheem A. Saddik and Ahmed A. K. Mohammed equally contributed to this work.
*Corresponding authors: A. A. Saddik (abdelreheem@aun.edu.eg) and O. Younis (osamayounis@sci.nvu.edu.eg)

Table of Contents

1. Table of Contents 1

2. Materials and Methods 2

3. Synthetic Procedures 2

4. Supplementary Figures 6

5. ^{1}H, ^{13}C NMR, HRMS, and FT-IR Spectra 20

6. References 35

2. Materials and Methods:

4-Bromobenzophenone, 4,4’-dimethoxybenzophenone, and diphenylmethane were purchased from Alfa Aesar. 4-Formylphenylboronic acid pinacol ester and *p*-nitrobenzyl cyanide were purchased from ThermoFisher Scientific. Anhydrous grade solvents were purchased from Sigma-Aldrich and Acros Organics. The purity of the compounds was confirmed using ^{1}H NMR, ^{13}C NMR, and MS techniques. NMR spectra of the tetraphenylethene compounds were recorded at VARIAN AS500 MHz in deuterated solvents. Coupling constants (J) are denoted in Hz and chemical shifts (δ) in ppm. Multiplicities are denoted as follows: s = singlet, d = doublet, t = triplet, m = multiplet, br = broad. High-resolution mass spectra (HRMS) were recorded on a Micromass Q-TOF MS spectrometer.

3. Synthetic Procedures:

Preparation of (2-(4-bromophenyl)ethene-1,1,2-triyl)tribenzene (5): (*Prepared according to literature*)[1]

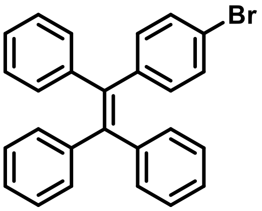

Compound **5** was synthesized with minor modifications following the documented procedure. In summary, Diphenylmethane was dissolved in tetrahydrofuran under a nitrogen atmosphere and cooled to 0°C. Slowly, 1.2 equivalents of n-butyl lithium (2.5M in hexanes) were added dropwise to the reaction mixture using a syringe or cannula. The resulting mixture was stirred at the same temperature for 30 minutes, causing it to turn dark brown. Then, 1.1 equivalents of Bromo-benzophenone were added gradually to the solution and stirred at room temperature for 12 hours. The reaction was quenched by adding ice-cold saturated ammonium chloride solution and then extracted with ethyl acetate twice. The organic layer was washed with excess water and brine solution, followed by drying with magnesium sulfate. The solution was filtered and evaporated to obtain the crude product. To purify the crude product, it was suspended in toluene and heated to reflux using a Dean-Stark apparatus for 3 hours while adding 0.5 equivalents of para-toluene sulfonic acid. The reaction mixture was then treated with water and extracted with ethyl acetate twice. The organic layer was washed with excess water and brine solution, dried with magnesium

sulfate, filtered, and evaporated to obtain the crude product. The pure product was isolated by recrystallization using methanol, resulting in an off-white crystalline solid with a yield of 80%.

Preparation of 4-(1,2,2-triphenylvinyl)benzaldehyde (A1): (*Prepared according to literature*)[2]

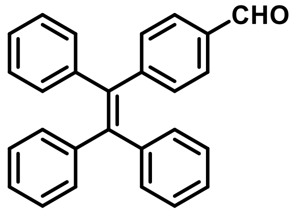

To a stirred solution of **5** (2.00 g, 4.08 mmol) in 50 mL anhydrous THF was added *n*-BuLi (6.4 ml, 2.5 M in hexane, 10.2 mmol) at -78°C under nitrogen. The mixture was first stirred for 2 h at this temperature and then warmed to room temperature. After stirring for 1 h, the mixture was cooled again to -78°C, and N,N-dimethylformamide (1.1 mL, 14.56 mmol) was added in one portion. The solution was stirred overnight and allowed to warm to room temperature gradually. 100 mL of 2 M aqueous hydrochloric acid solution was added to quench the reaction. After stirring for 2 h, the organic layer was separated, and the aqueous layer was extracted with 100 mL DCM three times. The organic layer was dried over $MgSO_4$. After solvent evaporation under reduced pressure, the residue was purified by silica gel column chromatography using hexane/ethyl acetate (4:1 v/v) as eluent to give the desired product as a pale yellow solid in 65% yield (1.1 g). ^{1}H NMR (300 MHz, $CDCl_3$, 25°C) δ= 7.00-7.05 (m, 6H; 6CH), 7.10-7.15 (m, 9H; 9CH), 7.21 (d, *J*=5 Hz, 2H; 2CH), 7.62 (d, *J*= 10 Hz, 2H; 2CH), 9.90 (s, 1H; 1CHO).

Preparation of 4,4'-(2-phenylethene-1,1-diyl)bis(methoxybenzene) (6): (*Prepared according to literature*) [3]

Step 1:

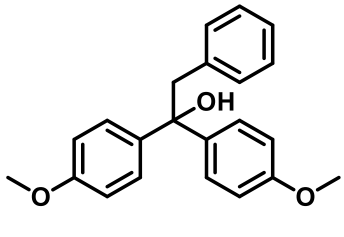

A dry three-neck RB flask was filled with magnesium (1.4 g, 62.5 mmol) under a nitrogen atmosphere. Then, 5 mL of tetrahydrofuran (THF) with a small amount of iodine as a catalyst was added and stirred at room temperature for 5 minutes. After that, a solution of 4-methoxyphenyl

bromide (9.0 g, 48.1 mmol) in 40 mL of THF was added to the suspension, and the mixture was refluxed for 3 h. The resulting freshly prepared Grignard solution was combined with a solution of methyl phenylacetate (3.0 g, 19.9 mmol) in THF (15 mL) at 0°C. The reaction mixture was then stirred for 24 h at room temperature. After closely monitoring the reaction progress using thin-layer chromatography (TLC), the reaction was quenched by adding a saturated aqueous solution of NH_4Cl (50 mL) at 0°C. The aqueous phase was extracted with ethyl acetate (50 mL x 3) and the combined organic layer was washed with a brine solution (50 mL) and dried with $MgSO_4$. The organic phase was evaporated, and the remaining residue was purified using silica gel column chromatography with an eluent mixture of 0-20% ethyl acetate and hexane. This process resulted in the formation of 1,1-bis(4-methoxyphenyl)-2-phenylethanol (3.5 g, 70%) as a white solid. ^{1}H NMR (300 MHz, $CDCl_3$, 25°C) δ= 2.23 (s, 1H; OH), 3.57 (s, 2H; CH_2), 3.77 (s, 6H; $2CH_3$), 6.81 (d, J = 8.6 Hz, 4H; 4CH), 6.89 (d, J = 3.6 Hz, 2H; 2CH), 7.10-7.15 (m, 3H; 3CH), 7.29 (d, J = 8.6 Hz, 4H; 4CH).

Step 2:

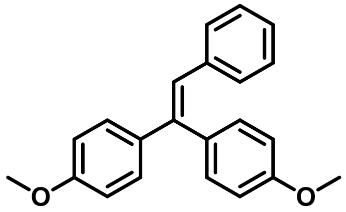

A mixture of 1,1-bis(4-methoxyphenyl)-2-phenylethanol (3.2 g, 9.58 mmol) and *p*-TsOH·H_2O (0.54 g, 2.87 mmol) in benzene (25 mL) was heated using a Dean-Stark apparatus under reflux conditions for 2 h. Water was then added, and the resulting reaction mixture was extracted with ethyl acetate (20 mL x 3). The combined organic layer was washed with brine (15 mL) and dried using $MgSO_4$. The solvent was evaporated, and the remaining residue was purified through column chromatography, using a mixture of ethyl acetate and hexane (0.5:9.5) as the eluent. As a result, 1,1-bis(4-methoxyphenyl)-2-phenylethylene (2.9 g, 95%) was obtained as a pale-yellow viscous oil. ^{1}H NMR (300 MHz, $CDCl_3$, 25°C) δ= 3.80 (s, 3H; CH_3), 3.81 (s, 3H; CH_3), 6.80-6.85 (m, 5H; 5CH), 7.00-7.15 (m, 7H; 7CH), 7.46 (d, J = 8.8 Hz, 2H; 2CH).

Preparation of 4,4'-(2-bromo-2-phenylethene-1,1-diyl)bis(methoxybenzene) (7): (*Prepared according to literature*) [3]

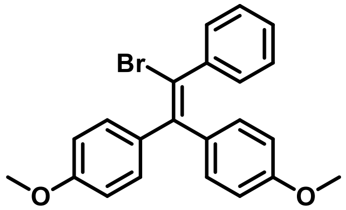

A solution of **6** (2.8 g, 8.86 mmol) in dichloromethane (20 mL) was subjected to the addition of bromine (0.5 mL, 9.74 mmol) at 0°C and stirred for 1 h at this temperature. Then, the reaction was allowed to reach room temperature for 2 h to achieve complete conversion. To quench the reaction, an organic base, Et_3N (5 mL), was added at 0°C. The resulting reaction mixture was extracted with ethyl acetate (20 mL x 3). The combined organic layer was washed with brine (25 mL) and dried using $MgSO_4$. The solvent was then evaporated, and the remaining residue was purified by column chromatography using a mixture of ethyl acetate and hexane (1:9) as the eluent. The pure 2,2-bis(4-methoxyphenyl)-1-phenylvinyl bromide (**7**) was yielded as a pale yellow solid (3.1 g, 90%). ^{1}H NMR (300 MHz, $CDCl_3$, 25°C) δ= 3.68 (s, 3H; CH_3), 3.82 (s, 3H; CH_3), 6.58 (d, J = 8.8 Hz, 2H; 2CH), 6.84 (d, J = 8.8 Hz, 2H; 2CH), 6.89 (d, J = 8.8 Hz, 2H; 2CH), 7.10-7.20 (m, 3H; 3CH), 7.25-7.35 (m, 4H; 4CH).

Preparation of 4-(2,2-bis(4-methoxyphenyl)-1-phenylvinyl)benzaldehyde (A2): (*Prepared according to literature*)[4]

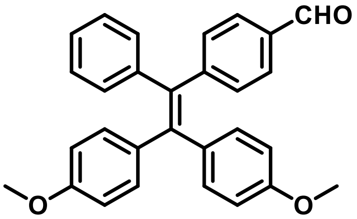

A solution of compound **7** (1.5 g, 3.8 mmol) and 4-Formylphenylboronic acid pinacol ester (0.96 g, 4.15 mmol) was dissolved in a THF-water (8:2) solvent system, with a total volume of 30 mL. Potassium carbonate (0.75 g, 5.7 mmol) was added to the solution and degassed for 30 minutes under a nitrogen atmosphere. Tetrakis(triphenylphosphine)palladium $Pd(PPh_3)_4$ (0.4 g, 0.38 mmol) was then added and degassed for an additional 10 minutes. The reaction mixture was heated to reflux under nitrogen for 16 h. Once the reaction was determined to be complete by TLC, the mixture was filtered and washed with THF. Water was added, and the resulting mixture was extracted with ethyl acetate twice. The combined organic layer was washed with brine (25 mL) and dried using $MgSO_4$. The solvent was evaporated, and the remaining residue was purified using

column chromatography with an eluent mixture ranging from 0% to 50% ethyl acetate and hexane. The pure material of compound **A2** was yielded as a yellow solid material (1.3 g, 81%). ^{1}H NMR (300 MHz, $CDCl_3$, 25°C) δ= 3.74 (s, 3H; CH_3), 3.75 (s, 3H; CH_3), 6.64 (dd, J = 2.5, 7.5 Hz, 4H; 4CH), 6.93 (dd, J = 5, 5 Hz, 4H; 4CH), 7.00 (dd, J = 2.5, 7.5 Hz, 2H; 2CH), 7.61 (d, J= 10 Hz, 2H; 2CH), 9.90 (s, 1H; CHO).

4. Supplementary Figures

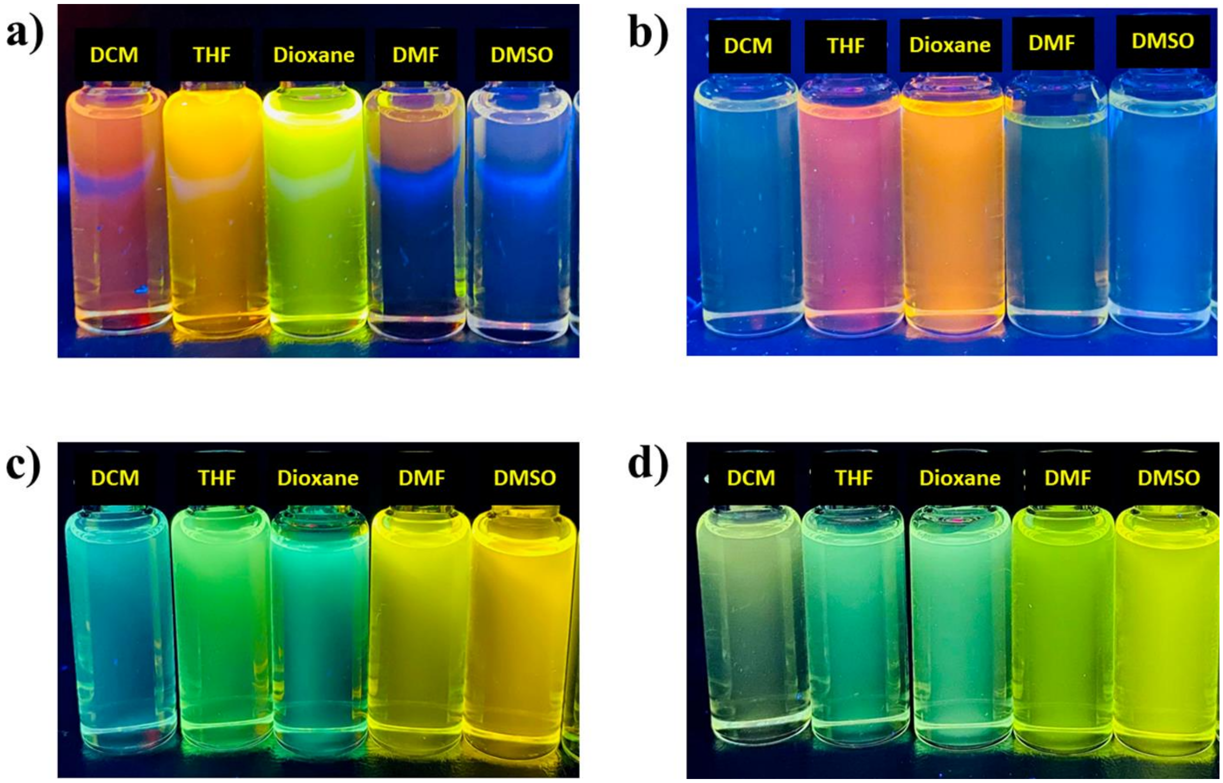

Figure S1. Effect of different organic solvents on; a) compound **1**, b) compound **2**, c) compound **3**, and d) compound **4**.

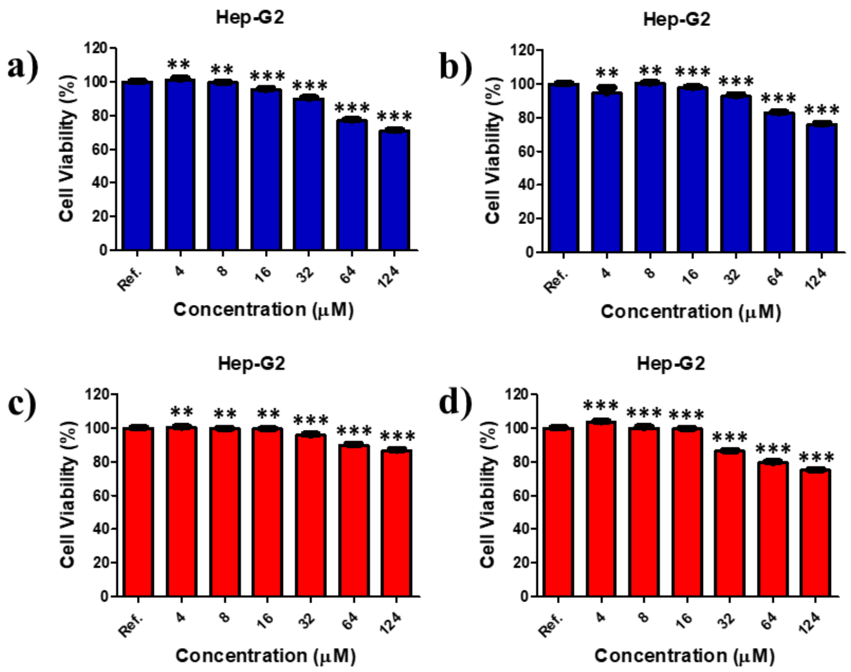

Figure S2. Cytotoxicity data results obtained from the MTT assay for; a) compound **1**, b) compound **2**, c) compound **3**, and d) compound **4** against **Hep-G2** cells. ** means *P* value > 0.05. *** means *P* value < 0.05.

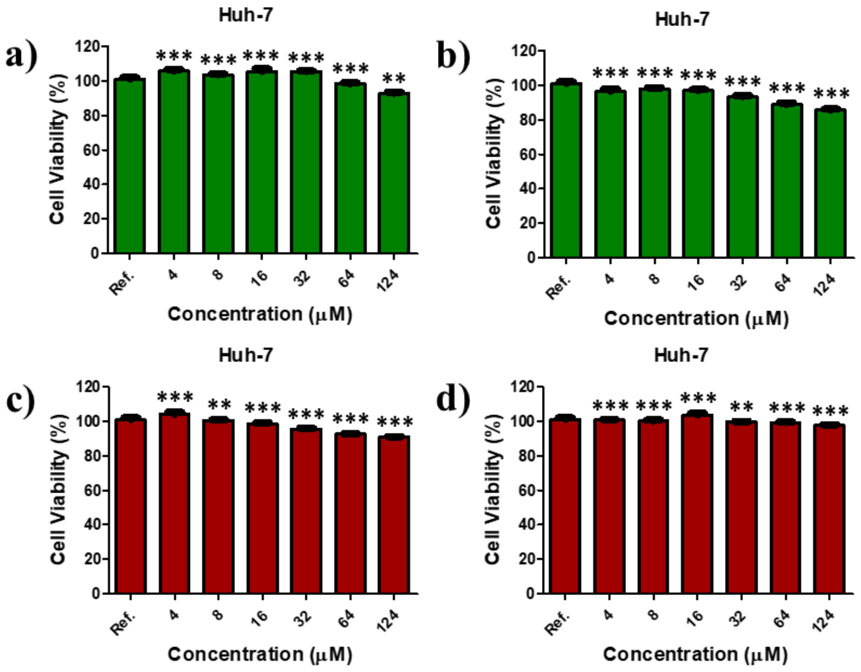

Figure S3. Cytotoxicity data results obtained from the MTT assay for; a) compound **1**, b) compound **2**, c) compound **3**, and d) compound **4** against **Huh-7** cells. ** means *P* value > 0.05. *** means *P* value < 0.05.

Table S1: The Cartesian coordinates (Z-Matrix) of optimized geometries of the ground state of compound 1.

E = -1608.174175 H

Charge = 0 Multiplicity = 1

Symbolic Z-Matrix:

C	-4.48669442	-4.56271705	-0.09559913
C	-4.97375594	-3.61553276	-0.99864649
C	-3.47566478	-4.20725186	0.79880946
C	-4.46219549	-2.32055277	-1.00007884
H	-5.75232450	-3.88680298	-1.70607879
C	-2.95810307	-2.91360591	0.79115598
H	-3.08815540	-4.93846082	1.50273557
C	-3.45490395	-1.94659591	-0.09712312
H	-4.84510874	-1.58441593	-1.69907154
C	-1.41095974	-0.49203406	-0.09599040
C	-0.64986693	-1.35911765	-0.89897171
C	-0.72110591	0.40731492	0.73883502
C	0.73449635	-1.29668897	-0.89393563
C	0.66245354	0.46279231	0.75925439
H	-1.28852642	1.07310750	1.37906383
C	1.42950515	-0.38942063	-0.06534777
H	1.30177772	-1.96335473	-1.53871998
H	1.14556243	1.17092182	1.41893201
H	-2.16733083	-2.64075861	1.48383893
H	-1.15767129	-2.07565966	-1.53630756
C	-2.89253899	-0.56816357	-0.10131462
C	-3.68272620	0.54591569	-0.11873957
C	-3.13715686	1.89969771	-0.40204480
C	-2.27843923	2.12188078	-1.49006861
C	-3.49932330	2.99302975	0.40128663
C	-1.77264549	3.39297756	-1.74932302
H	-2.00977824	1.28774848	-2.12995466
C	-2.98406367	4.26234712	0.14922094
H	-4.18031399	2.83624664	1.23229625
C	-2.11700053	4.46665034	-0.92597762
H	-1.11123579	3.54664203	-2.59733029
H	-3.26107222	5.09360353	0.79139330
C	-5.14696332	0.48994347	0.13079661
C	-6.03093505	1.21799685	-0.68201827
C	-5.67581219	-0.25100364	1.19994799
C	-7.40485996	1.17773660	-0.45506740
H	-5.63171881	1.81095518	-1.49923659
C	-7.04760245	-0.28163956	1.43400481
H	-5.00193847	-0.80417253	1.84523133
C	-7.91860825	0.42685553	0.60351665
H	-8.07446555	1.73648139	-1.10294903
H	-7.43760740	-0.85702584	2.26885887
H	-8.98926231	0.40009275	0.78527175
H	-4.88786631	-5.57219759	-0.09337556

H	-1.71882696	5.45734993	-1.12546245
C	2.87783084	-0.41264364	-0.13729768
C	3.83683281	0.24084496	0.58075376
H	3.25883851	-1.09577594	-0.89215075
C	3.51586098	1.09858572	1.67967183
N	3.31106193	1.79733586	2.58841886
C	5.28362845	0.07706693	0.30479969
C	6.23003423	0.31122422	1.31964024
C	5.74600395	-0.30834156	-0.96788930
C	7.58795925	0.13786096	1.08634652
H	5.89466043	0.62643240	2.30186710
C	7.09968042	-0.48886680	-1.21283531
H	5.04426389	-0.43933167	-1.78409359
C	8.00691438	-0.26729764	-0.17835253
H	8.32208812	0.30716579	1.86366962
H	7.46547192	-0.78016879	-2.18913646
N	9.43847638	-0.45264155	-0.43354558
O	9.77615173	-0.81030913	-1.56279810
O	10.21736380	-0.24181177	0.49630855

Table S2: The Cartesian coordinates (Z-Matrix) of optimized geometries of the ground state of compound 2.

E = -1837.235021 H

Charge = 0 Multiplicity = 1

Symbolic Z-Matrix:

C	3.81184584	-4.92843030	0.58945237
C	4.28923919	-3.91958089	1.42902947
C	2.82482599	-4.63152473	-0.35171481
C	3.79156074	-2.62381018	1.32156343
H	5.04848479	-4.14374153	2.17319023
C	2.32248266	-3.33565372	-0.45410335
H	2.44410588	-5.41011605	-1.00702512
C	2.80867078	-2.30814716	0.37006789
H	4.16688891	-1.84044181	1.97169114
C	0.78431652	-0.83507950	0.21032765
C	-0.01194381	-1.64715373	1.03787616
C	0.12732635	0.02482948	-0.69184768
C	-1.39437095	-1.56834625	0.99079685
C	-1.25347832	0.09651792	-0.75326909
H	0.72107019	0.64640329	-1.35224667
C	-2.05521038	-0.69913470	0.09558913
H	-1.98785483	-2.19109355	1.65574367
H	-1.70889223	0.77245178	-1.46443748
H	1.55078909	-3.10871251	-1.18394528
H	0.46804814	-2.33311498	1.72817698
C	2.26242743	-0.92759170	0.25890642
C	3.06854903	0.17829181	0.21383503
C	2.53679698	1.54868339	0.41342050

C	1.63309357	1.84148817	1.45243066
C	2.93960292	2.60277039	-0.41703024
C	1.13318897	3.12027545	1.63151302
H	1.32480587	1.04763112	2.12449819
C	2.43613779	3.89319608	-0.25851978
H	3.65141605	2.40624255	-1.21291779
C	1.52330432	4.15682964	0.76977170
H	0.44065847	3.34833296	2.43510407
H	2.75957582	4.67672858	-0.93321401
C	4.52946122	0.08519698	-0.02268656
C	5.42967984	0.86725149	0.72590425
C	5.06093942	-0.75158115	-1.01290664
C	6.79779697	0.78740380	0.51869605
H	5.04217084	1.53712024	1.48713452
C	6.43229303	-0.83462859	-1.24207639
H	4.38827529	-1.35157629	-1.61607582
C	7.31123289	-0.06779685	-0.46724771
H	7.49344656	1.37831346	1.10558693
H	6.80068654	-1.49010779	-2.02205031
H	4.20122525	-5.93913486	0.67304883
C	-3.50368312	-0.69954634	0.12899558
C	-4.43502871	-0.07052036	-0.64669669
H	-3.91437556	-1.33854488	0.90665601
C	-4.07347162	0.72602632	-1.77823834
N	-3.83509896	1.37529074	-2.71523284
C	-5.89015062	-0.20155700	-0.40155805
C	-6.80732523	-0.00006284	-1.45005605
C	-6.39136371	-0.52297361	0.87447988
C	-8.17308615	-0.14375108	-1.24501892
H	-6.44228654	0.26589989	-2.43626372
C	-7.75330285	-0.67353759	1.09156736
H	-5.71306251	-0.62688362	1.71403227
C	-8.63078086	-0.48608576	0.02497341
H	-8.88431646	0.00067917	-2.04822678
H	-8.14807855	-0.91566481	2.07001755
N	-10.07037942	-0.64012243	0.25027076
O	-10.44273224	-0.94239641	1.38500891
O	-10.82238273	-0.46042442	-0.70813077
O	8.66710941	-0.07528678	-0.59875588
O	0.97102085	5.37526592	1.02151519
C	9.23576376	-0.92847281	-1.57915134
H	8.89885576	-0.66081344	-2.58909619
H	10.31546379	-0.78769227	-1.50888937
H	8.99178209	-1.98107835	-1.38558363
C	1.32285840	6.45517349	0.17089616
H	1.03743206	6.25503140	-0.86984582
H	0.76773816	7.31921595	0.53906713
H	2.39898370	6.66741643	0.21567751

Table S3: The Cartesian coordinates (Z-Matrix) of optimized geometries of the ground state of compound 3.

E = -1459.025516 H

Charge = 0 Multiplicity = 1

Symbolic Z-Matrix:

C	-4.02184192	-4.51866412	-0.14715676
C	-4.51082190	-3.54492223	-1.02040372
C	-2.98264666	-4.19990027	0.72867339
C	-3.97355327	-2.26042406	-1.00983154
H	-5.31105820	-3.78725455	-1.71419355
C	-2.43936723	-2.91685079	0.73241027
H	-2.59254190	-4.95198924	1.40892202
C	-2.93800268	-1.92278946	-0.12455085
H	-4.35778657	-1.50395614	-1.68595525
C	-0.86367320	-0.51068543	-0.12913777
C	-0.13038680	-1.36553046	-0.96882293
C	-0.14446136	0.35057065	0.71871789
C	1.25571833	-1.32839757	-0.98300924
C	1.24130033	0.38044444	0.71841966
H	-0.68911993	1.00970757	1.38562225
C	1.98165967	-0.46098494	-0.13948506
H	1.80051595	-1.98648378	-1.65559729
H	1.74694622	1.06262060	1.38859421
H	-1.62550133	-2.67278335	1.40868882
H	-0.66012414	-2.05361096	-1.62012622
C	-2.34732838	-0.55629458	-0.11575098
C	-3.11542051	0.57253458	-0.10223401
C	-2.54862842	1.92218820	-0.36582750
C	-1.69567631	2.15055910	-1.45712799
C	-2.88666019	3.00739703	0.45854893
C	-1.17355065	3.41823183	-1.69966931
H	-1.44195988	1.32287533	-2.11114939
C	-2.35596926	4.27362819	0.22257196
H	-3.56216855	2.84683525	1.29335412
C	-1.49556032	4.48359886	-0.85658249
H	-0.51554281	3.57551149	-2.54974209
H	-2.61540368	5.09779983	0.88120271
C	-4.57720745	0.54061371	0.16939629
C	-5.46091193	1.29242277	-0.62171814
C	-5.10339700	-0.20157000	1.23882507
C	-6.83178777	1.27416850	-0.37408614
H	-5.06341582	1.88736623	-1.43841597
C	-6.47206758	-0.21159148	1.49316550
H	-4.42934402	-0.77324463	1.86768352
C	-7.34317694	0.52082752	0.68396180
H	-7.50100231	1.85185928	-1.00575454
H	-6.85931488	-0.78955092	2.32766252
H	-4.44291550	-5.52010020	-0.15415170
C	3.43204307	-0.51064252	-0.23175743

C	4.41237144	0.08484760	0.50526619
H	3.79041922	-1.15812022	-1.02823555
C	4.11252744	0.87943905	1.65886566
N	3.92828229	1.52167178	2.61276663
C	5.85330046	-0.06076825	0.19962488
C	6.82434860	0.10788311	1.20173185
C	6.30577294	-0.37195850	-1.09440408
C	8.17610791	-0.05689019	0.93586380
H	6.51225256	0.36403513	2.20945049
C	7.65474237	-0.54067705	-1.36856674
H	5.59408637	-0.45871575	-1.90940770
C	8.61814501	-0.39104221	-0.35504616
H	8.90276654	0.07713755	1.73336708
H	7.97561101	-0.76685624	-2.38250649
N	9.97547269	-0.50102413	-0.64083218
H	10.57129331	-0.70633664	0.15082972
H	10.20698383	-1.06624389	-1.44752857
H	-1.08423508	5.47153850	-1.04310654
H	-8.41136477	0.51080025	0.88170651

Table S4: The Cartesian coordinates (Z-Matrix) of optimized geometries of the ground state of compound 4.

E = -1688.085798 H

Charge = 0 Multiplicity = 1

Symbolic Z-Matrix:

C	3.39902994	-4.88876401	0.61619167
C	3.87035091	-3.86229332	1.43762719
C	2.38816095	-4.62096548	-0.30830916
C	3.34357540	-2.57826022	1.32785586
H	4.64776024	-4.06350619	2.16965043
C	1.85646170	-3.33702799	-0.41238036
H	2.01095218	-5.41349691	-0.94894043
C	2.33683508	-2.29130247	0.39217307
H	3.71404782	-1.78148370	1.96424611
C	0.27620944	-0.86706976	0.24071986
C	-0.49467919	-1.67055039	1.09807820
C	-0.40720657	-0.04602974	-0.67519462
C	-1.88000340	-1.62192218	1.06261476
C	-1.79136684	-0.00448022	-0.72348014
H	0.16646507	0.57249706	-1.35648430
C	-2.56898962	-0.79376824	0.15131968
H	-2.45356317	-2.23928297	1.74987521
H	-2.26764674	0.64556106	-1.44520202
H	1.06482586	-3.13295211	-1.12728595
H	0.00569461	-2.32746001	1.80262168
C	1.75820150	-0.92430968	0.27828338
C	2.53789413	0.19797596	0.21711701
C	1.97815008	1.56052422	0.40065364

C	1.07210576	1.84935409	1.43853739
C	2.35935159	2.61218714	-0.44233587
C	0.55126736	3.12160615	1.60553805
H	0.77617171	1.05695542	2.11768099
C	1.83545567	3.89632982	-0.29548711
H	3.07202285	2.41947901	-1.23844922
C	0.92203151	4.15547144	0.73263040
H	-0.14477144	3.34551233	2.40732303
H	2.14397218	4.67773858	-0.97967997
C	4.00016447	0.13538173	-0.02960668
C	4.88971437	0.93345196	0.71420523
C	4.54170279	-0.68800438	-1.02496447
C	6.25812111	0.88108068	0.49803389
H	4.49362526	1.59504263	1.47837156
C	5.91353427	-0.74479434	-1.26238001
H	3.87664634	-1.30019478	-1.62444729
C	6.78192901	0.03752565	-0.49204194
H	6.94565952	1.48507217	1.08131085
H	6.28953678	-1.39232066	-2.04545865
H	3.81104679	-5.89042733	0.70146704
C	-4.02141942	-0.82587300	0.19631618
C	-4.97075378	-0.26033505	-0.60278850
H	-4.41339048	-1.42835951	1.01216346
C	-4.62576585	0.47104741	-1.78488620
N	-4.40463802	1.06155389	-2.76418024
C	-6.42243361	-0.37813802	-0.33745928
C	-7.35876385	-0.25984135	-1.37891687
C	-6.91963352	-0.61212883	0.95632694
C	-8.72000914	-0.39981310	-1.14892040
H	-7.01156473	-0.06334919	-2.38857610
C	-8.27842597	-0.75585473	1.19480385
H	-6.23547296	-0.65723176	1.79784921
C	-9.20678755	-0.65727201	0.14332897
H	-9.41912939	-0.30614288	-1.97630039
H	-8.63413325	-0.92195455	2.20877536
O	8.13838323	0.05660980	-0.63211605
O	0.35028349	5.36875878	0.97403662
C	8.71529714	-0.78487231	-1.61667174
H	8.36675470	-0.52321521	-2.62442107
H	9.79295443	-0.62489033	-1.55419870
H	8.49194386	-1.84220080	-1.42302677
C	0.67775362	6.44175350	0.10664735
H	0.39072323	6.22201037	-0.92985720
H	0.10932348	7.30139464	0.46519398
H	1.75023816	6.67445893	0.14150831
N	-10.57433968	-0.74142098	0.38992983
H	-11.14409392	-0.99026624	-0.40850025
H	-10.83544579	-1.26097994	1.21810374

Table S5: The Cartesian coordinates (Z-Matrix) of optimized geometries of the most stable excited state of compound 1.

E = -1608.097687 H

Charge = 0 Multiplicity = 1

Symbolic Z-Matrix:

C	-5.29897300	-4.02784200	-0.93049600
C	-5.49528600	-2.81353500	-1.59754300
C	-4.31420200	-4.13568600	0.05644200
C	-4.72834500	-1.70656600	-1.26695200
H	-6.24427700	-2.73671800	-2.37905000
C	-3.52466200	-3.03851500	0.37301900
H	-4.16359100	-5.07673500	0.57524400
C	-3.72998300	-1.79558600	-0.26892700
H	-4.87177400	-0.77150200	-1.79496800
C	-1.48136800	-0.91662500	0.38260300
C	-0.75402300	-1.82555500	-0.41390700
C	-0.80112600	-0.21337800	1.39796400
C	0.61964500	-1.94847100	-0.25772000
C	0.56638600	-0.36820900	1.56135100
H	-1.35266300	0.46378000	2.04118300
C	1.31088300	-1.20020000	0.70834600
H	1.18518000	-2.59504200	-0.92010100
H	1.08833100	0.19370300	2.32856100
H	-2.76222600	-3.11563500	1.13972400
H	-1.26200900	-2.36811800	-1.20430500
C	-2.88964600	-0.65302300	0.08022900
C	-3.40258800	0.66624700	0.09492600
C	-2.53391200	1.83404800	-0.00506200
C	-1.34313600	1.82180200	-0.77608000
C	-2.87955500	3.02667800	0.67968800
C	-0.53053700	2.94184500	-0.84614300
H	-1.05976200	0.93267300	-1.32452500
C	-2.05718000	4.14102100	0.61530700
H	-3.77176800	3.04529100	1.29532700
C	-0.88293200	4.10167600	-0.14644100
H	0.38957500	2.88714400	-1.41620300
H	-2.31799100	5.03696800	1.16951500
C	-4.84477600	0.90389800	0.22821900
C	-5.47061700	1.92040500	-0.52599600
C	-5.63181900	0.13984300	1.11722400
C	-6.83824800	2.14083800	-0.41654100
H	-4.87670700	2.50622700	-1.21908000
C	-6.99305100	0.38094000	1.24094800
H	-5.15902500	-0.62620400	1.72125800
C	-7.60239400	1.37509200	0.46854000
H	-7.31074300	2.90821800	-1.02133000
H	-7.58249100	-0.20261600	1.94088400
H	-8.66931300	1.55398500	0.55869900
H	-5.90810500	-4.88973300	-1.18443600

H	-0.23639800	4.97258000	-0.18764200
C	2.79590700	-1.22955300	0.77566100
C	3.57053100	-0.36370400	0.04465200
H	3.25793600	-1.96554400	1.42294800
C	2.84286000	0.55459000	-0.79496400
N	2.21261400	1.27442700	-1.45863400
C	5.02114100	-0.27231500	0.02503900
C	5.69116100	0.64384000	-0.82338100
C	5.82352400	-1.10018700	0.85410800
C	7.06781300	0.73315400	-0.85120100
H	5.10906200	1.29222000	-1.47191100
C	7.19847400	-1.02124500	0.83651000
H	5.35390600	-1.81152000	1.52685100
C	7.84257000	-0.10214900	-0.01888500
H	7.58149000	1.43108100	-1.49874200
H	7.81484200	-1.64773200	1.46803400
N	9.24976000	-0.02030300	-0.03645300
O	9.90777700	-0.78751500	0.72565700
O	9.79180900	0.81788100	-0.81360100

Table S6: The Cartesian coordinates (Z-Matrix) of optimized geometries of the most stable excited state of compound 2.

E = -1837.117123 H

Charge = 0 Multiplicity = 1

Symbolic Z-Matrix:

C	4.04211800	-4.85250200	0.64773200
C	4.50447500	-3.80091400	1.44083300
C	3.00340500	-4.62740900	-0.26028500
C	3.94338400	-2.53253000	1.31944400
H	5.30153700	-3.96905200	2.15943800
C	2.43606100	-3.36057100	-0.37597100
H	2.63271500	-5.44175100	-0.87630700
C	2.90629600	-2.28874600	0.40057400
H	4.30270800	-1.71733400	1.93779300
C	0.80883300	-0.92471000	0.21309900
C	0.03394100	-1.74278300	1.07175000
C	0.12178600	-0.11352600	-0.71799700
C	-1.34147800	-1.72829400	1.01646700
C	-1.25439200	-0.09382900	-0.78903100
H	0.69761100	0.51664400	-1.38685000
C	-2.04821900	-0.91027700	0.07952900
H	-1.91552200	-2.36099600	1.68827900
H	-1.73307100	0.55584900	-1.50907400
H	1.62601700	-3.19048900	-1.07885400
H	0.54133400	-2.38629100	1.78353300
C	2.28696900	-0.94325400	0.28148800
C	3.03768000	0.20490900	0.24096600
C	2.43803000	1.54872000	0.41945400
C	1.51627500	1.80703800	1.44963100

C	2.79704300	2.61559700	-0.42093500
C	0.95974700	3.06538400	1.61576700
H	1.23674100	1.00504700	2.12469700
C	2.24594000	3.88668000	-0.27075600
H	3.51804300	2.44158600	-1.21348100
C	1.31731200	4.11761700	0.75048200
H	0.24408300	3.26758500	2.40569300
H	2.54053500	4.67873500	-0.94845900
C	4.50155800	0.18619200	0.00129200
C	5.36761900	0.99258500	0.76547000
C	5.06768300	-0.60520600	-1.00927800
C	6.73708100	0.97533100	0.55664700
H	4.95245700	1.62841100	1.54138600
C	6.44108200	-0.62817000	-1.23658800
H	4.41942900	-1.21764500	-1.62680700
C	7.28698100	0.16047500	-0.44588400
H	7.40780300	1.58232800	1.15587800
H	6.83735700	-1.25191700	-2.02875900
H	4.47946200	-5.84218200	0.74209500
C	-3.46671000	-0.95823000	0.09622900
C	-4.41467500	-0.38150300	-0.77503000
H	-3.88981700	-1.55198700	0.90038800
C	-4.01876400	0.17045000	-2.02290500
N	-3.72673100	0.62620300	-3.05836400
C	-5.83856500	-0.36109300	-0.48012300
C	-6.79143200	-0.07727900	-1.49299800
C	-6.32226900	-0.62966600	0.82964800
C	-8.14764000	-0.08361900	-1.22525300
H	-6.44824000	0.13530700	-2.50019500
C	-7.67732100	-0.63451500	1.10581500
H	-5.62336000	-0.79865100	1.64134800
C	-8.58975100	-0.37153800	0.07583800
H	-8.88133900	0.12476000	-1.99287100
H	-8.05582400	-0.82804700	2.10116700
N	-10.00518800	-0.38501300	0.36245600
O	-10.36154800	-0.67299100	1.52361500
O	-10.78766900	-0.10244900	-0.56742100
O	8.63999500	0.21308700	-0.57457700
O	0.70906000	5.30561200	0.99368000
C	9.24897500	-0.59655100	-1.56929600
H	8.90400000	-0.32317600	-2.57466500
H	10.32099000	-0.41059700	-1.49085500
H	9.04895200	-1.66160900	-1.39579200
C	1.00410800	6.40003800	0.13590400
H	0.72950200	6.17558600	-0.90216900
H	0.40386600	7.23431600	0.50023700
H	2.06776100	6.66537000	0.18180800

Table S7: The Cartesian coordinates (Z-Matrix) of optimized geometries of the most stable excited state of compound 3.

E = -1458.933062 H

Charge = 0 Multiplicity = 1

Symbolic Z-Matrix:

C	-4.55911300	-4.27861600	-0.55208900
C	-4.98375100	-3.12079700	-1.20993500
C	-3.40484600	-4.24068100	0.23571700
C	-4.26727700	-1.93793400	-1.07923400
H	-5.87765000	-3.14223200	-1.82688500
C	-2.68056000	-3.06094800	0.36133700
H	-3.07574400	-5.13220600	0.76223900
C	-3.09493300	-1.87996500	-0.29334000
H	-4.59838800	-1.04225300	-1.59313600
C	-0.91242700	-0.66814000	-0.04149800
C	-0.13705400	-1.68470800	-0.67531700
C	-0.18247200	0.36115500	0.62755000
C	1.23640000	-1.65385700	-0.65079600
C	1.19174300	0.38167500	0.66728800
H	-0.73471700	1.13699100	1.14511900
C	1.96478100	-0.62246600	0.01737000
H	1.79550800	-2.42854600	-1.17032700
H	1.68287700	1.17907400	1.20815000
H	-1.79978200	-3.02864100	0.99396600
H	-0.64526900	-2.46761600	-1.22787600
C	-2.34765600	-0.62544100	-0.14281400
C	-3.05539800	0.62465800	-0.11117600
C	-2.45446300	1.83066900	-0.66292300
C	-1.53418200	1.76892200	-1.74254500
C	-2.73696700	3.11130700	-0.12226300
C	-0.94622400	2.91728800	-2.25268100
H	-1.30470500	0.80485900	-2.18181200
C	-2.13622100	4.25435700	-0.63015100
H	-3.40517100	3.18664000	0.72869100
C	-1.23804100	4.16839300	-1.70066800
H	-0.25220500	2.83851700	-3.08483500
H	-2.35401400	5.21912700	-0.18036300
C	-4.38788800	0.69313500	0.47925300
C	-5.38067300	1.56561200	-0.03140700
C	-4.75034000	-0.14441700	1.56305900
C	-6.65963300	1.59193900	0.50781300
H	-5.13983300	2.19585300	-0.88095800
C	-6.02895900	-0.10848300	2.10310200
H	-4.00442700	-0.80890400	1.98449300
C	-6.99425100	0.75672700	1.57995300
H	-7.40645000	2.25804500	0.08429200
H	-6.27500100	-0.75544600	2.94066100
H	-5.12523400	-5.20066700	-0.64788400
C	3.39117300	-0.67963100	-0.03394100

C	4.37109000	0.13159700	0.51967200
H	3.76911100	-1.52694400	-0.59897800
C	4.04629500	1.22022500	1.37906600
N	3.83373500	2.11868300	2.09407300
C	5.80537700	-0.08023400	0.28621400
C	6.77334500	0.53703600	1.10878200
C	6.28678900	-0.89712800	-0.76030000
C	8.12951100	0.33436300	0.91768400
H	6.44642800	1.18043600	1.91920000
C	7.64180400	-1.10646300	-0.95585800
H	5.59083600	-1.35776200	-1.45301700
C	8.59418000	-0.49886300	-0.11645100
H	8.84559000	0.82403100	1.57331900
H	7.97755900	-1.73205200	-1.77961100
N	9.95433500	-0.66500200	-0.34386300
H	10.55983500	-0.48122200	0.44565100
H	10.22040700	-1.49980300	-0.84979200
H	-0.76513700	5.06429300	-2.09211200
H	-7.99587500	0.77895900	1.99952600

Table S8: The Cartesian coordinates (Z-Matrix) of optimized geometries of the most stable excited state of compound 4.

E = -1687.997441 H

Charge = 0 Multiplicity = 1

Symbolic Z-Matrix:

C	3.99065400	-4.57517400	1.29467700
C	4.39726900	-3.35196200	1.83617300
C	2.86993200	-4.62389700	0.46004700
C	3.69813800	-2.18907400	1.54075400
H	5.26091900	-3.30806000	2.49364900
C	2.16189900	-3.46368500	0.16993700
H	2.55294100	-5.56954400	0.02946300
C	2.56335000	-2.21636700	0.69825400
H	4.00964800	-1.24348700	1.96973100
C	0.38949500	-1.06919800	0.19622500
C	-0.42055100	-1.95100000	0.96613900
C	-0.29313900	-0.17740000	-0.67686900
C	-1.79369300	-1.91719100	0.87922300
C	-1.66607900	-0.15050500	-0.77534700
H	0.29181200	0.49001300	-1.30038600
C	-2.48096500	-1.01299200	0.01372500
H	-2.38315900	-2.58809800	1.49977900
H	-2.12348400	0.53873300	-1.47176700
H	1.30357500	-3.49875900	-0.49214600
H	0.05539000	-2.62844200	1.66820400
C	1.82401900	-0.99181200	0.37860500
C	2.51192500	0.25387200	0.25678000
C	1.84418000	1.51211600	0.56758400
C	0.84850200	1.59833300	1.57916300

C	2.14969500	2.70344200	-0.12937300
C	0.22142600	2.78924000	1.87620100
H	0.58384800	0.70572800	2.13399700
C	1.51264500	3.90564600	0.15012500
H	2.87845900	2.67046500	-0.93210400
C	0.54040500	3.95836000	1.16096000
H	-0.53343900	2.84935900	2.65321900
H	1.76079900	4.78581300	-0.43097600
C	3.89364300	0.28962200	-0.21519600
C	4.82317600	1.24292400	0.27421700
C	4.37497700	-0.64602400	-1.15852500
C	6.14178000	1.24531600	-0.13724200
H	4.49548000	1.96399300	1.01554800
C	5.69702600	-0.64894800	-1.58541700
H	3.68675700	-1.37314000	-1.57439800
C	6.59479700	0.29782900	-1.07318300
H	6.85486600	1.96094800	0.25918200
H	6.01566700	-1.38135600	-2.31759800
H	4.54225200	-5.48307200	1.52145100
C	-3.91031800	-1.04225400	0.01555800
C	-4.85135300	-0.31180300	-0.70116000
H	-4.32692100	-1.78267500	0.69216300
C	-4.47274300	0.62493200	-1.70294200
N	-4.21358200	1.40141500	-2.53740200
C	-6.29851500	-0.45949400	-0.49475200
C	-7.22297700	0.04266400	-1.43648400
C	-6.83744400	-1.10021800	0.64200300
C	-8.59044900	-0.10437500	-1.26891300
H	-6.85303400	0.55013800	-2.32163000
C	-8.20466600	-1.25330400	0.81356800
H	-6.17817500	-1.46425300	1.42259700
C	-9.11164300	-0.76297300	-0.14173400
H	-9.27069800	0.29416200	-2.01823200
H	-8.58360900	-1.74232500	1.70832900
O	7.90827000	0.38244200	-1.41097100
O	-0.14572300	5.07257700	1.51547000
C	8.42257400	-0.55993900	-2.34052400
H	7.92102500	-0.47676900	-3.31308600
H	9.48033000	-0.31928000	-2.45575200
H	8.31903100	-1.58663400	-1.96692800
C	0.09330700	6.27122200	0.79081800
H	-0.14522600	6.14651800	-0.27265400
H	-0.56907400	7.01992100	1.22709300
H	1.13536700	6.59939300	0.89447700
N	-10.48885700	-0.86645400	0.05714900
H	-11.04690900	-0.81752600	-0.78629300
H	-10.77943500	-1.63221300	0.65238900

5. ^{1}H, ^{13}C NMR, HRMS, and FT-IR spectra.

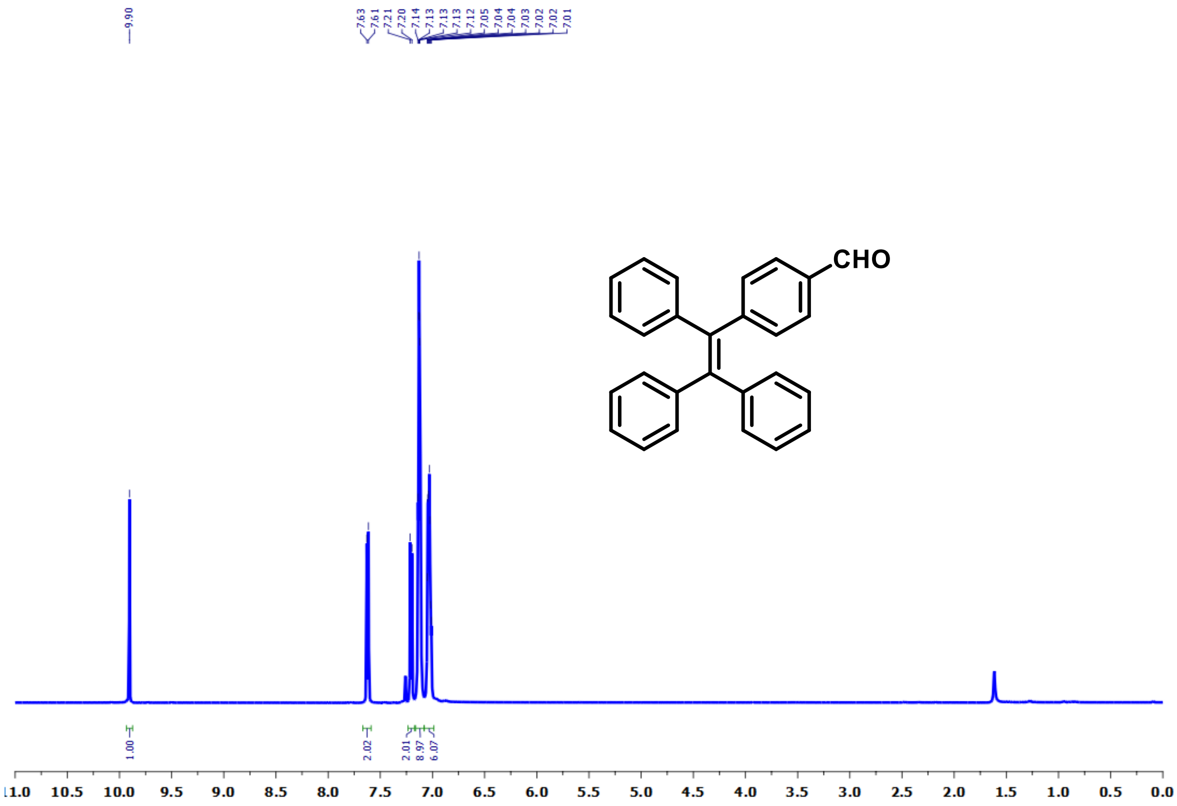

Figure S4. The ^{1}H-NMR spectra of compound **A1** in $CDCl_3$.

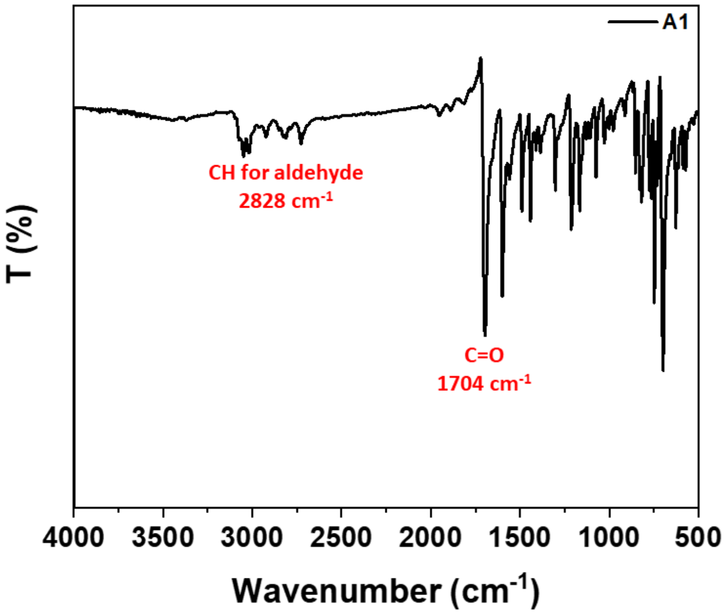

Figure S5. The FT-IR spectra of compound **A1**.

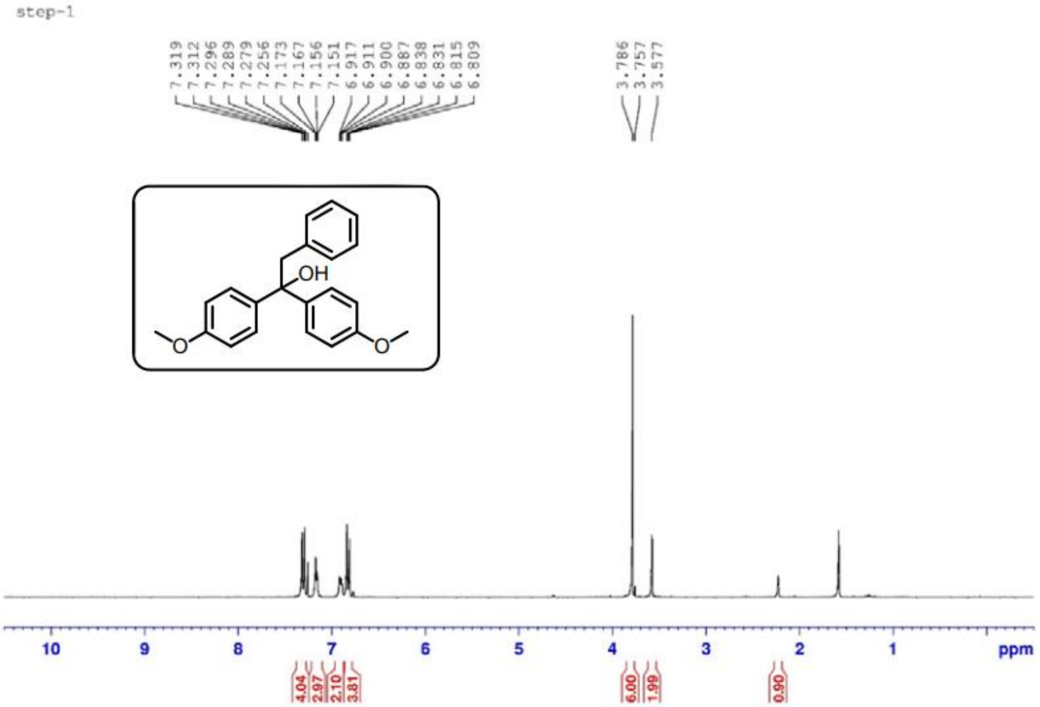

Figure S6. The ^{1}H-NMR spectra of **1,1-bis(4-methoxyphenyl)-2-phenylethanol** in $CDCl_3$.

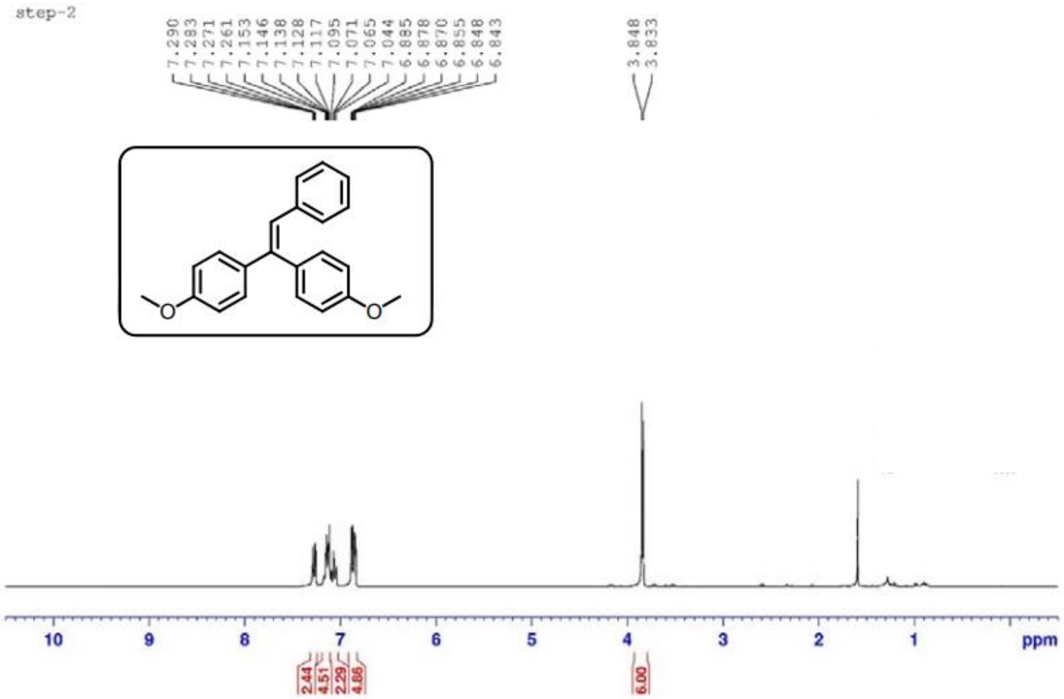

Figure S7. The ^{1}H-NMR spectra of compound **6** in $CDCl_3$.

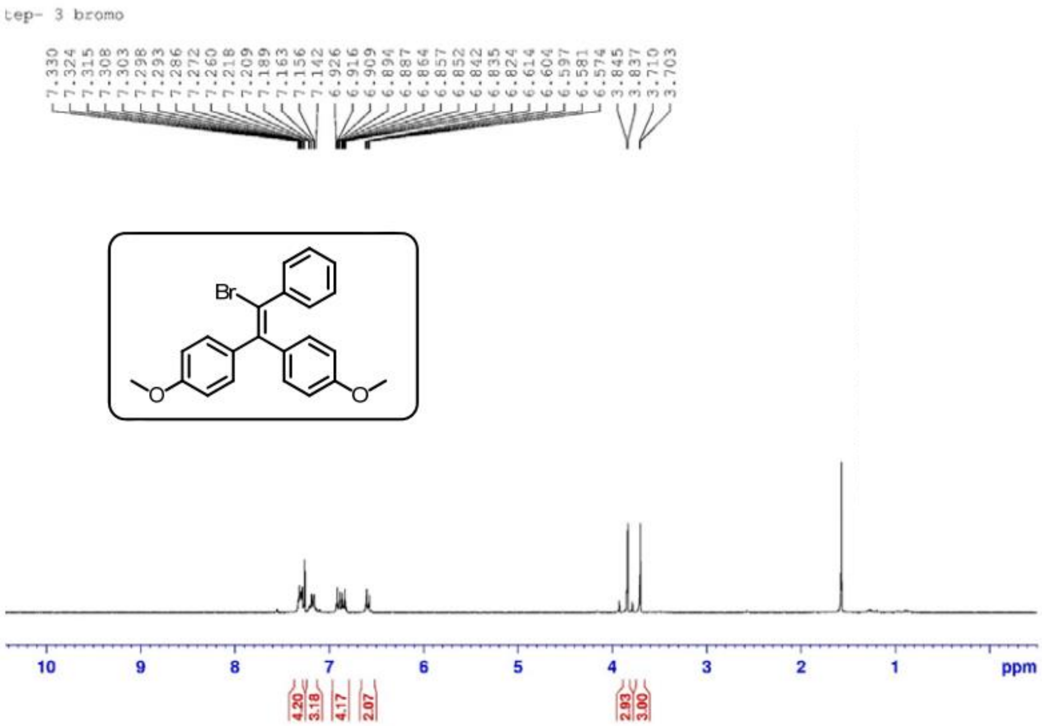

Figure S8. The ^{1}H-NMR spectra of compound **7** in $CDCl_3$.

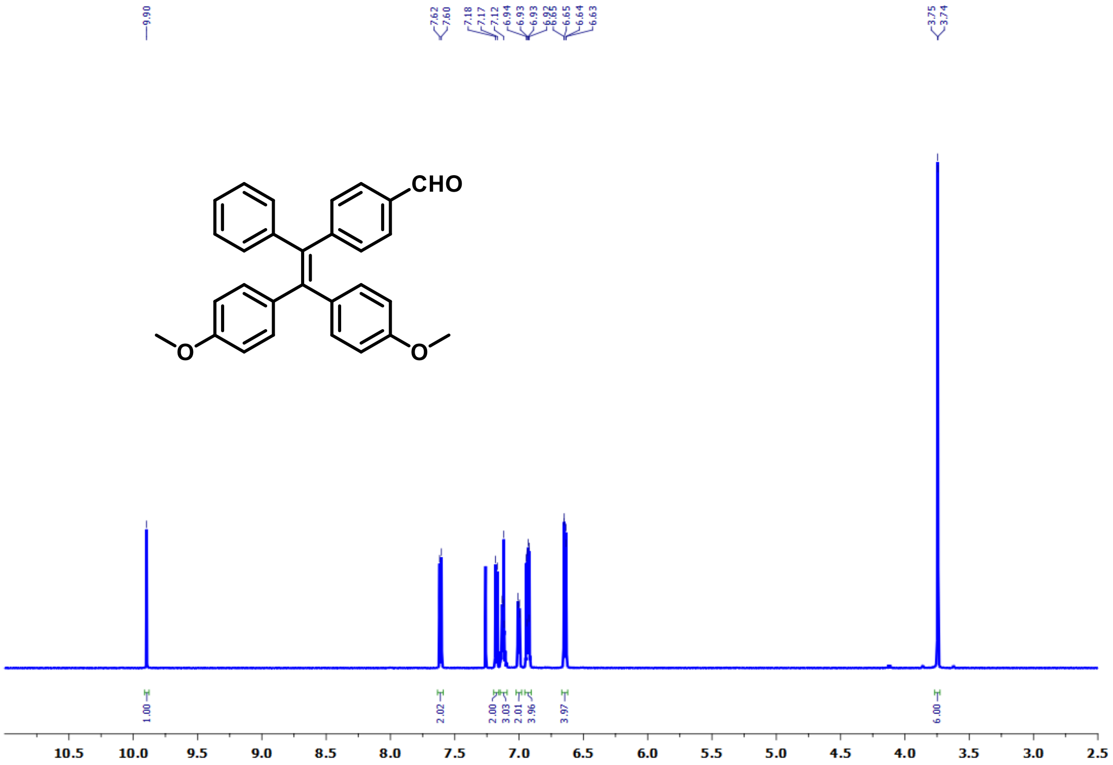

Figure S9. The ^{1}H-NMR spectra of compound **A2** in $CDCl_3$.

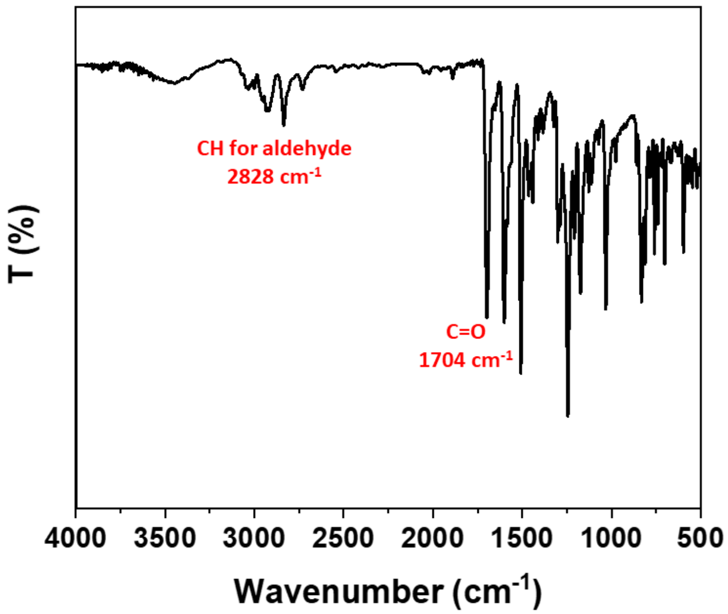

Figure S10. The FT-IR spectra of compound **A2**.

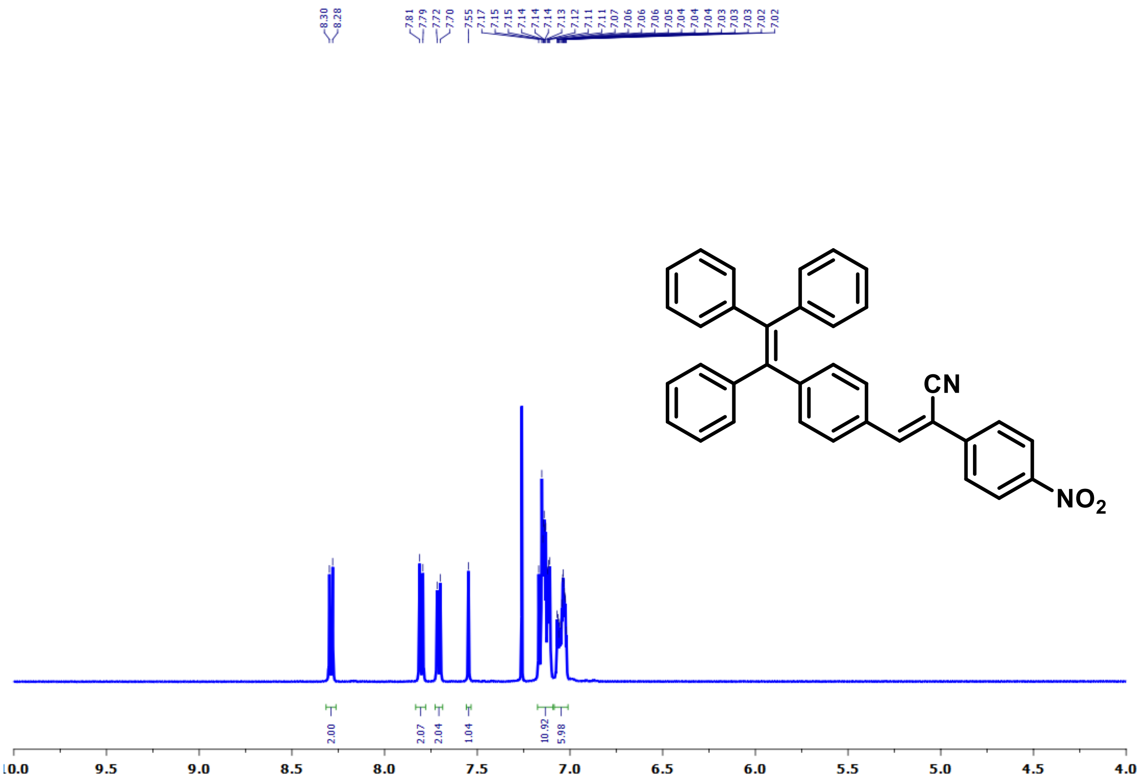

Figure S11. The ^{1}H-NMR spectra of compound **1** in $CDCl_3$.

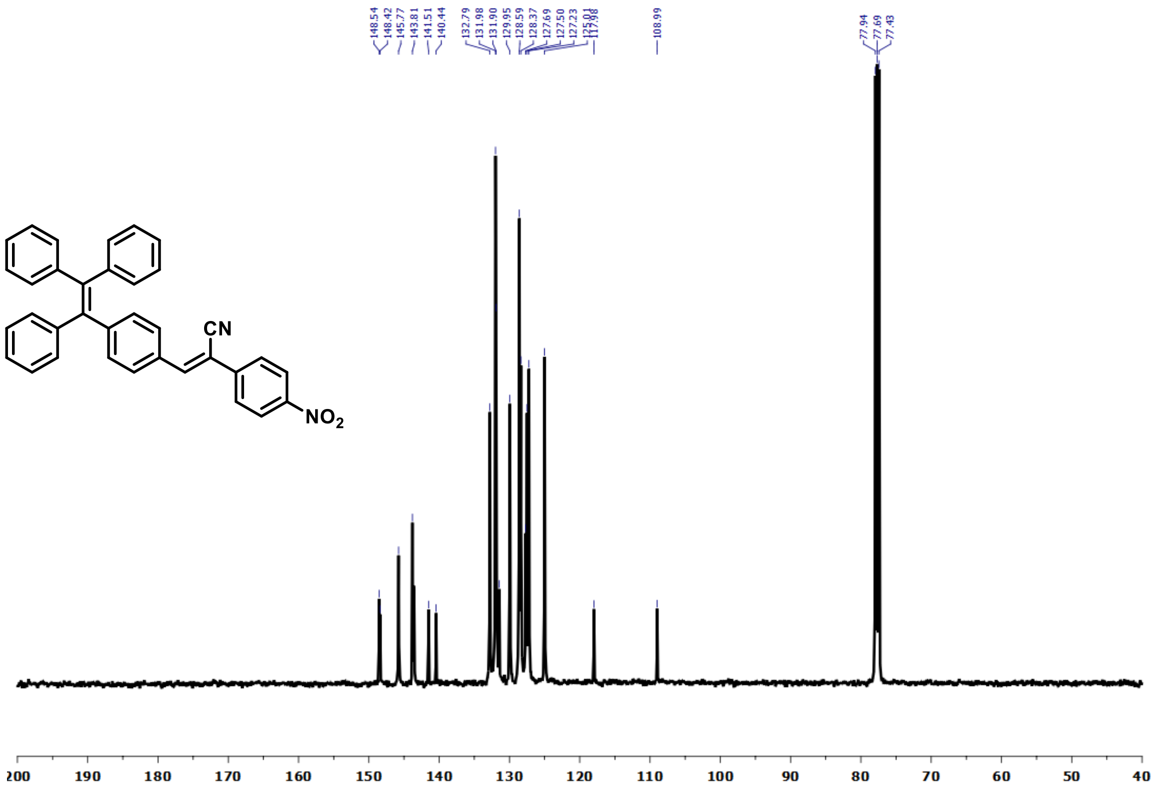

Figure S12. The ^{13}C-NMR spectra of compound **1** in $CDCl_3$.

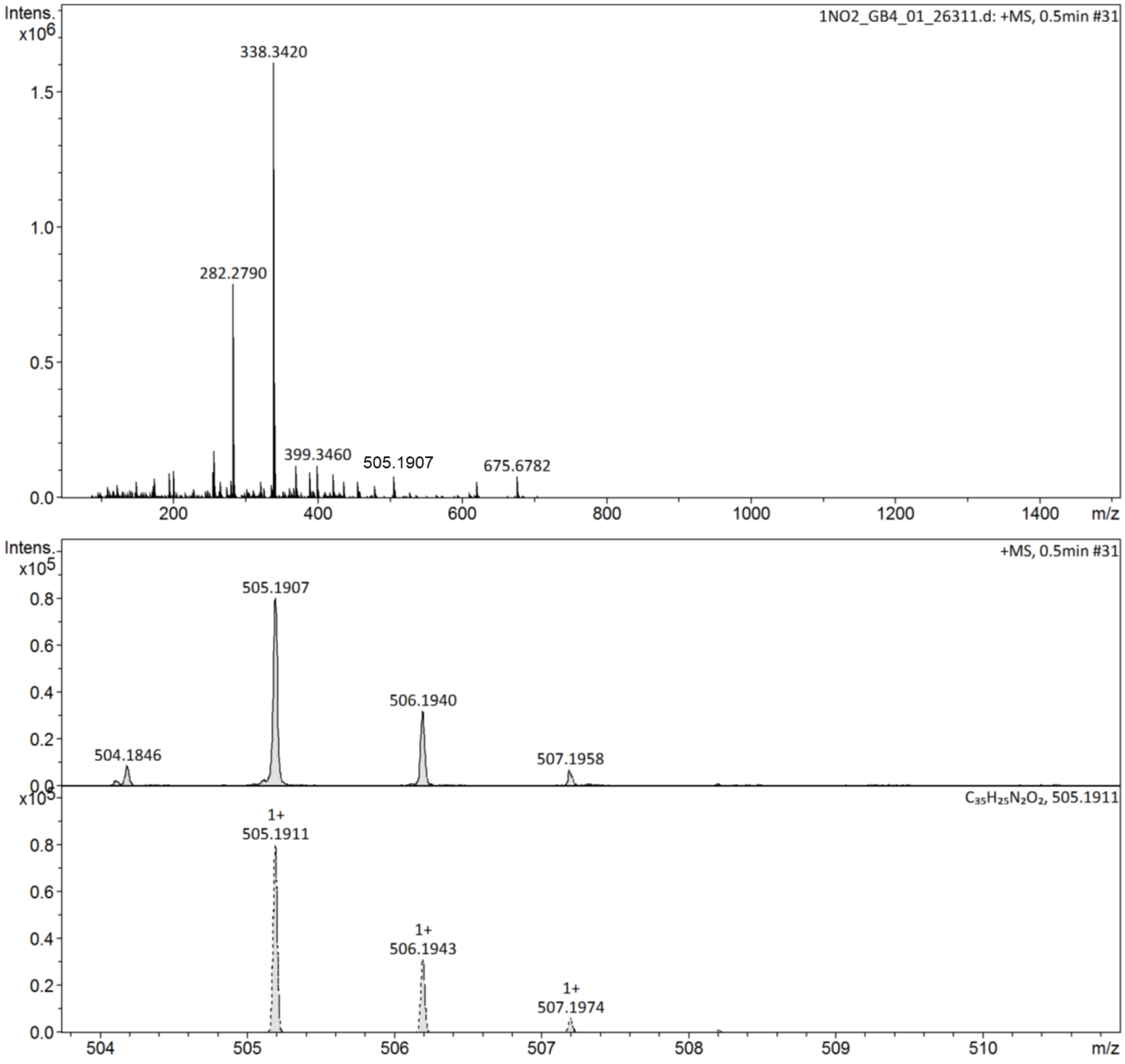

Figure S13. The HRMS of compound **1**.

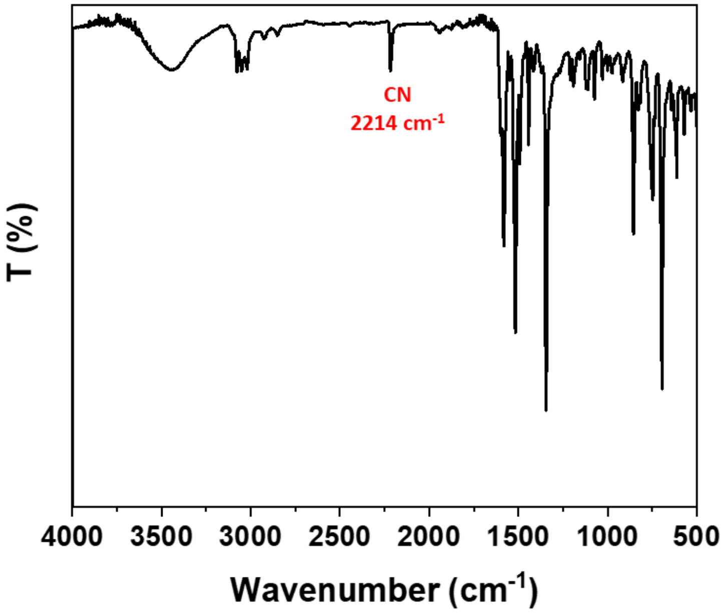

Figure S14. The FT-IR spectra of compound **1**.

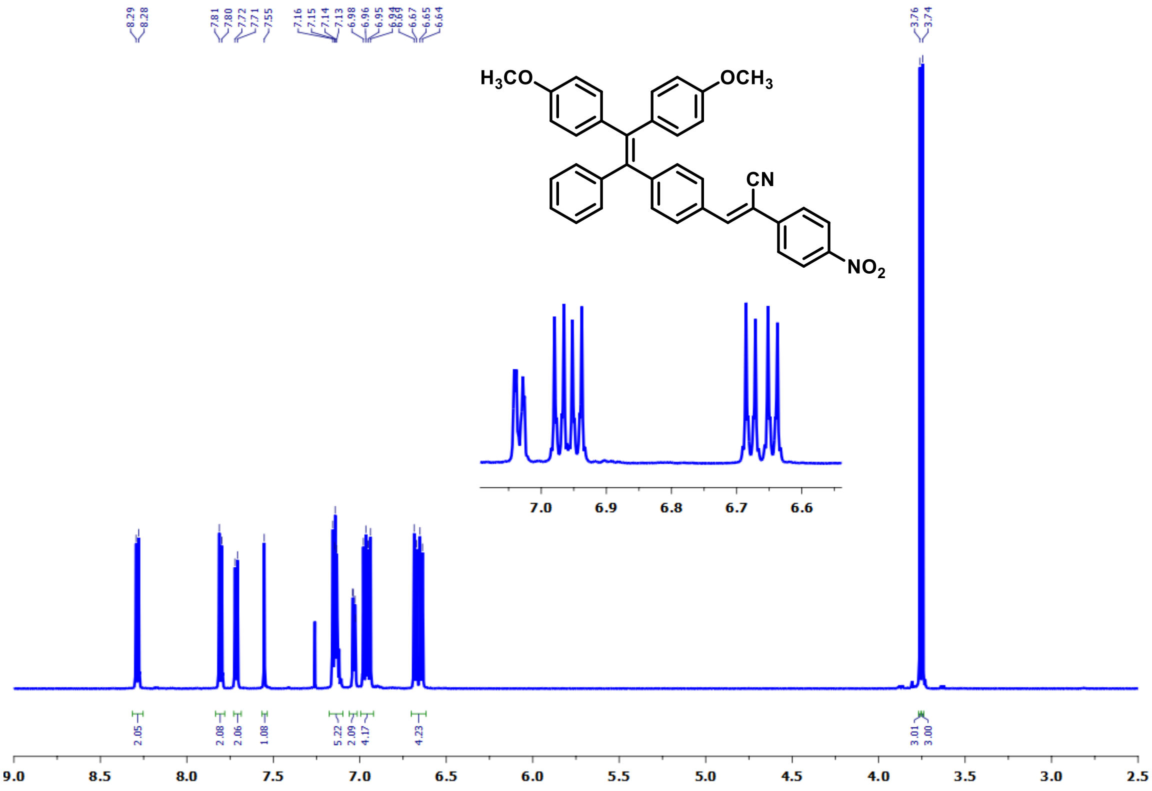

Figure S15. The ^{1}H-NMR spectra of compound **2** in $CDCl_3$.

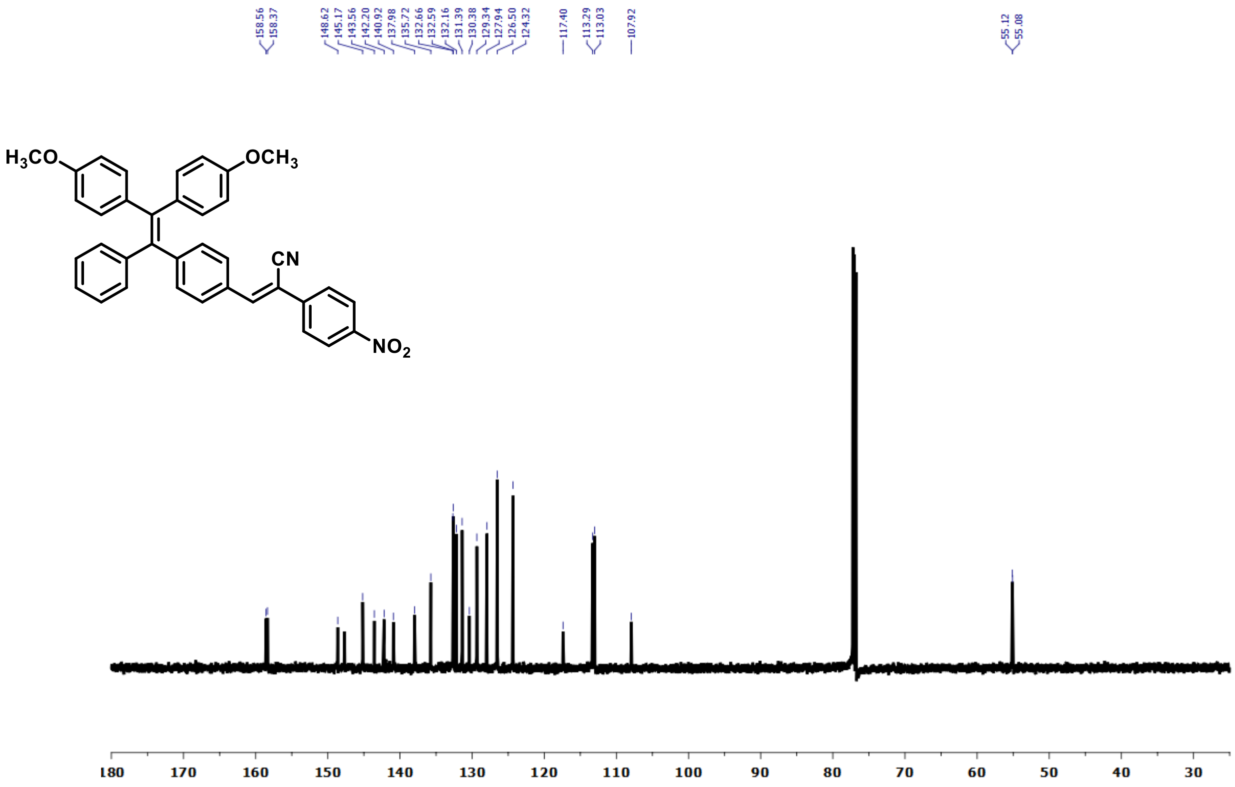

Figure S16. The ^{13}C-NMR spectra of compound **2** in $CDCl_3$.

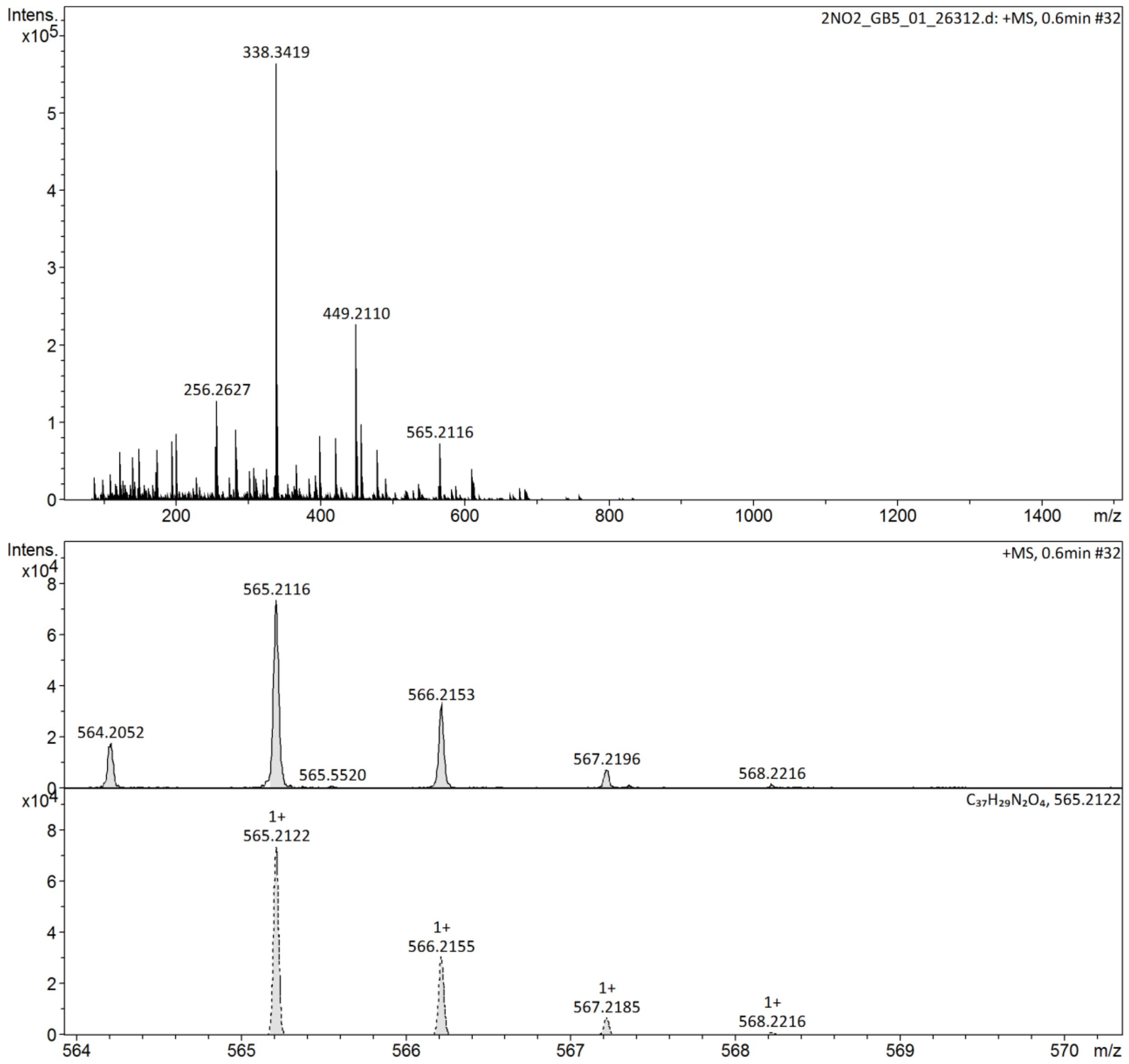

Figure S17. The HRMS of compound **2**.

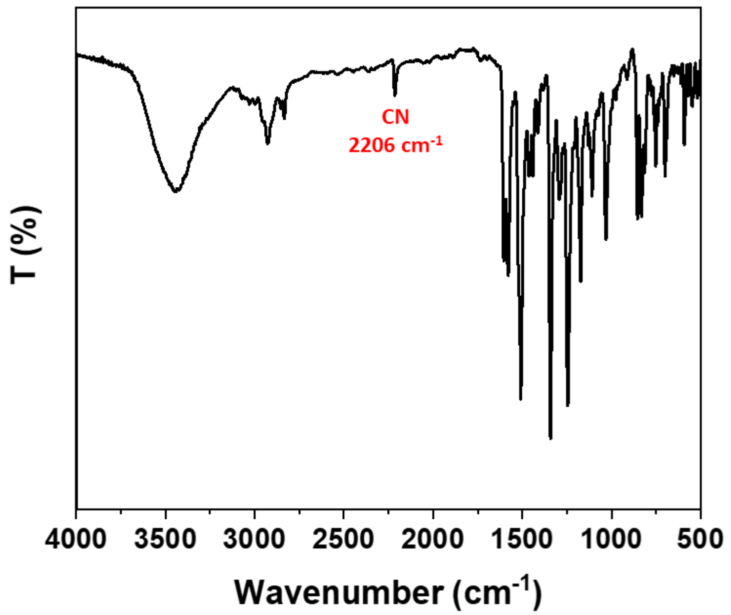

Figure S18. The FT-IR spectra of compound **2**.

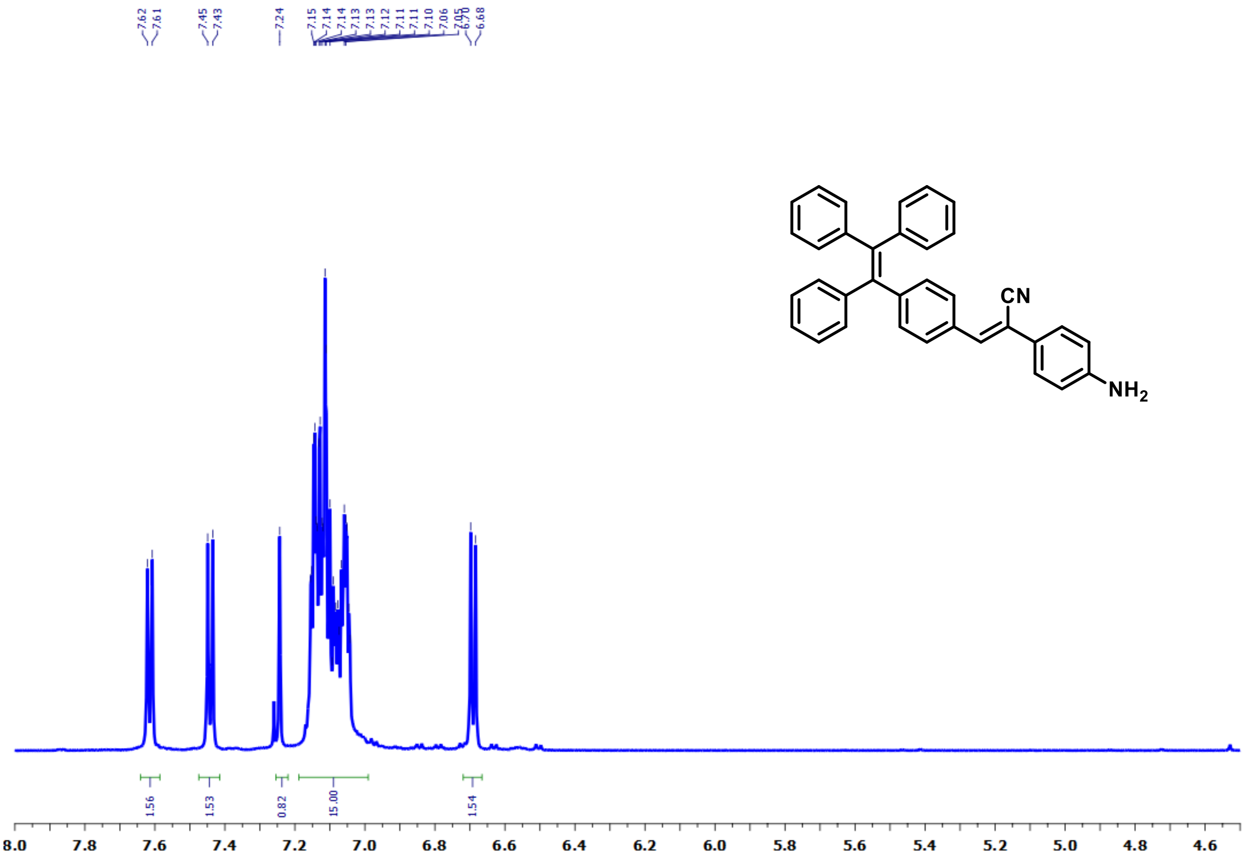

Figure S19. The ^{1}H-NMR of compound **3** in $CDCl_3$.

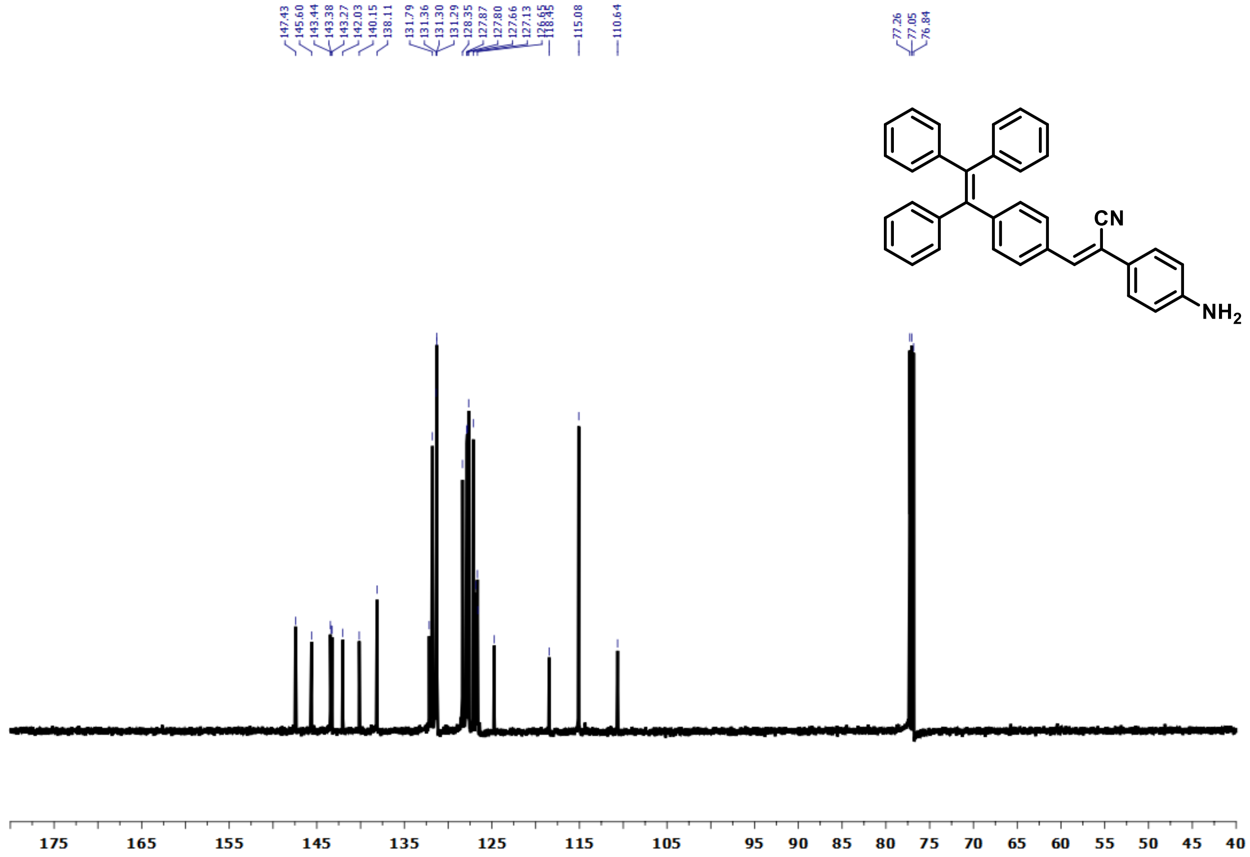

Figure S20. The ^{13}C-NMR of compound **3** in $CDCl_3$.

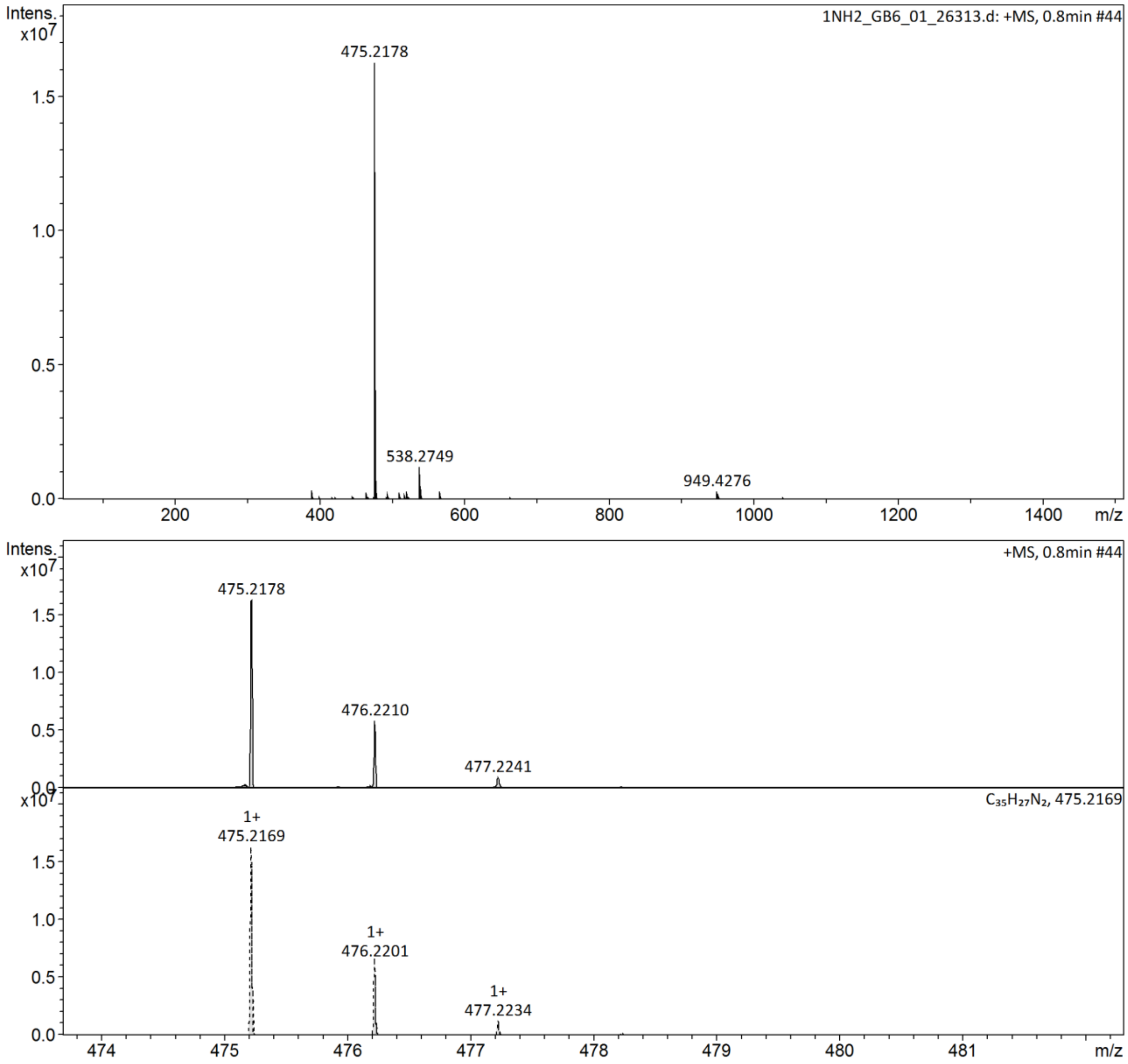

Figure S21. The HRMS of compound **3**.

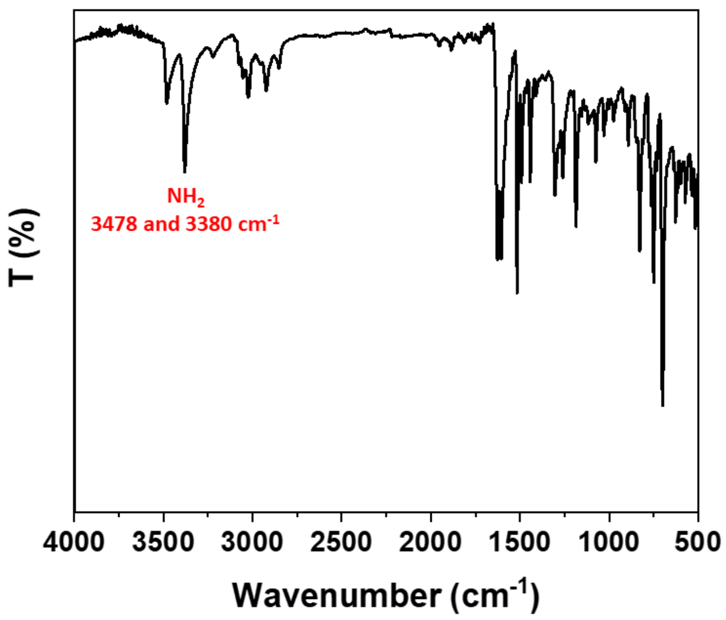

Figure S22. The FT-IR spectra of compound **3**.

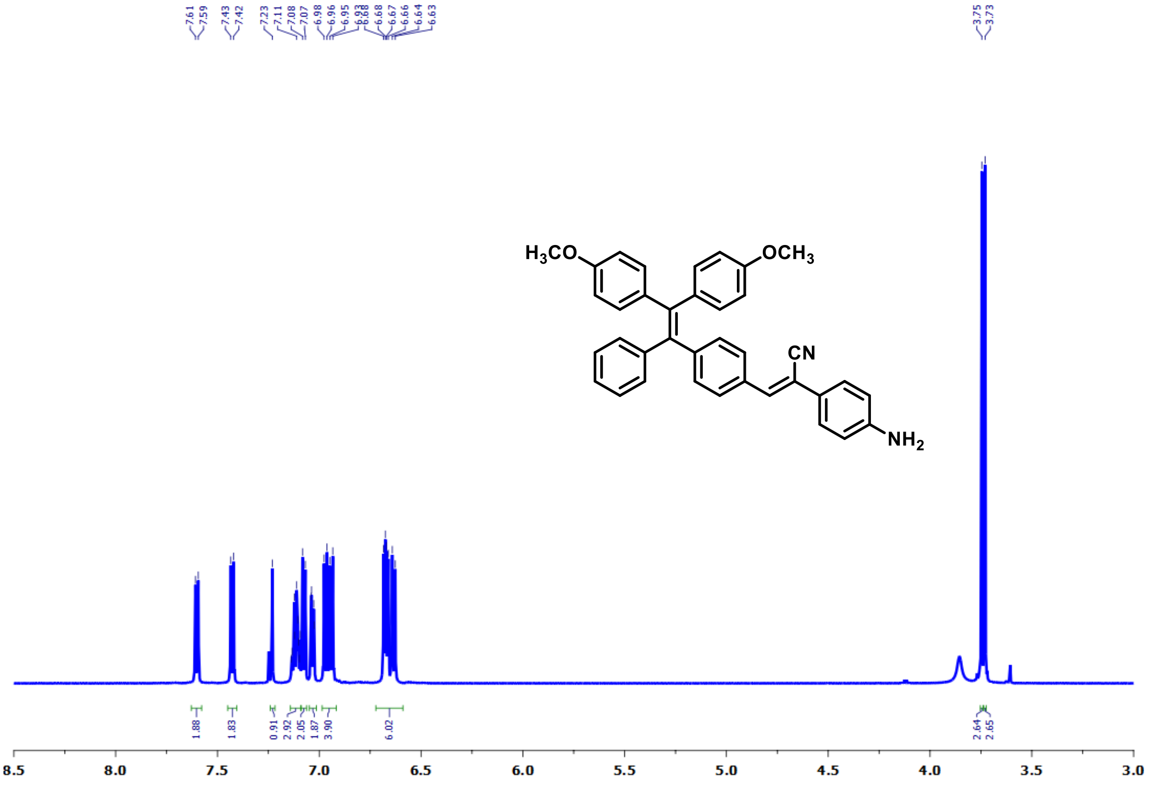

Figure S23. The ^{1}H-NMR of compound **4** in $CDCl_3$.

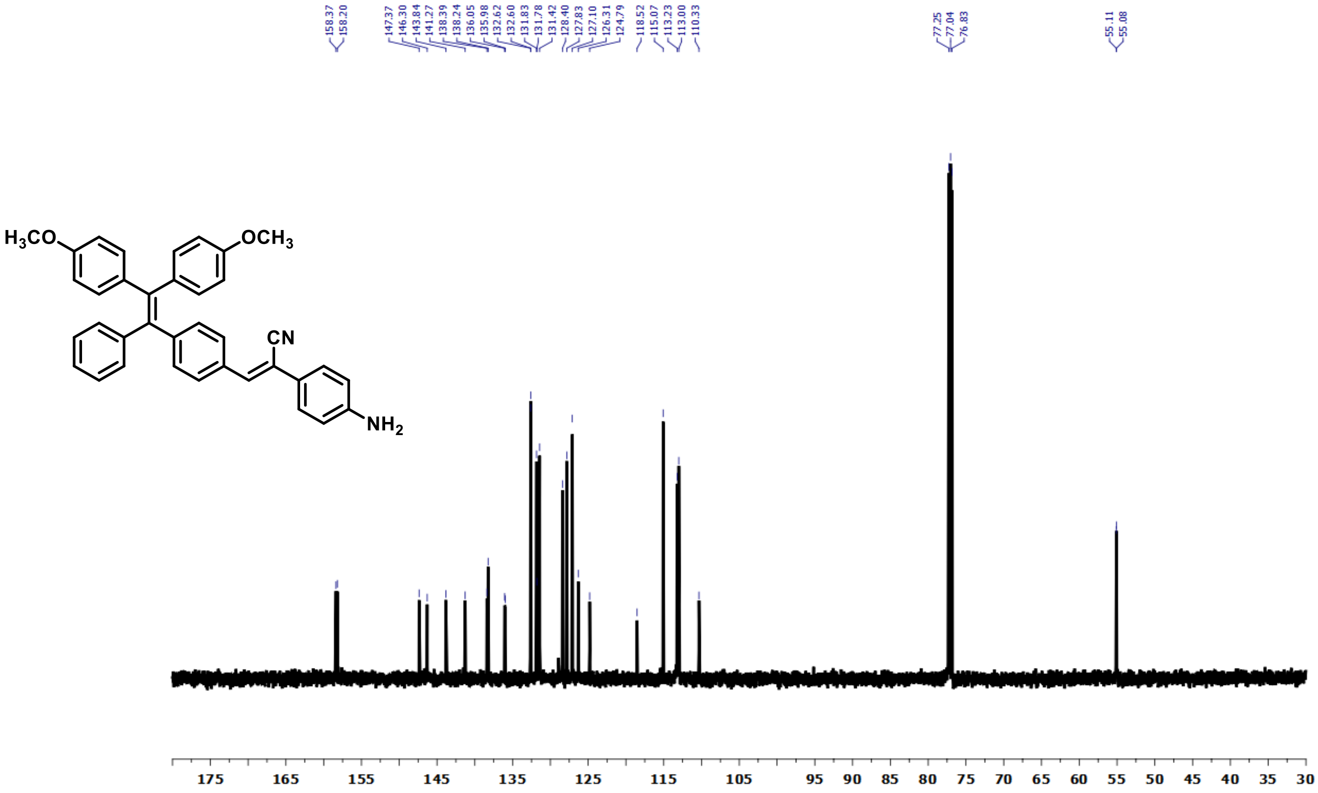

Figure S24. The ^{13}C-NMR of compound **4** in $CDCl_3$.

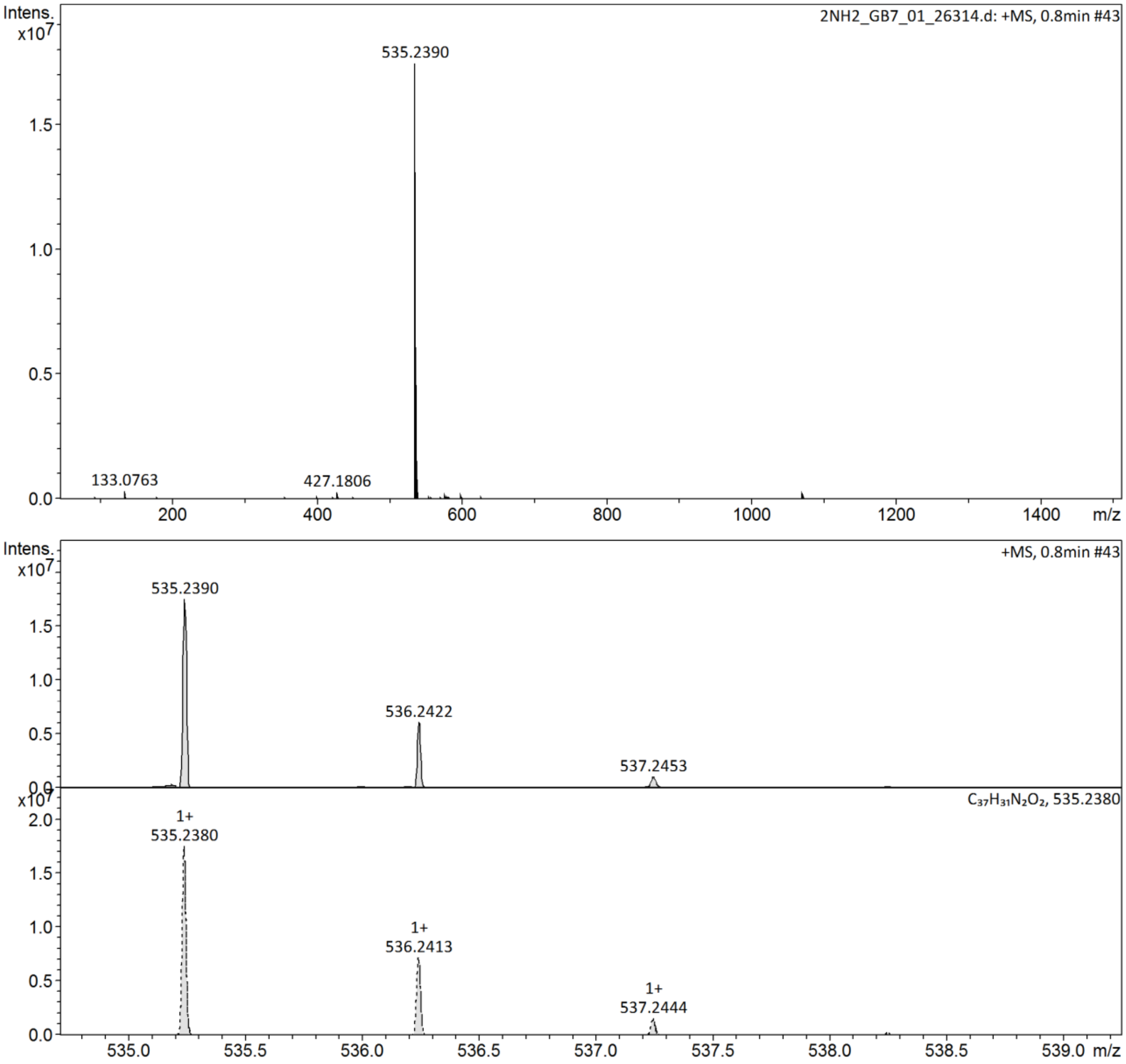

Figure S25. The HRMS of compound **4**.

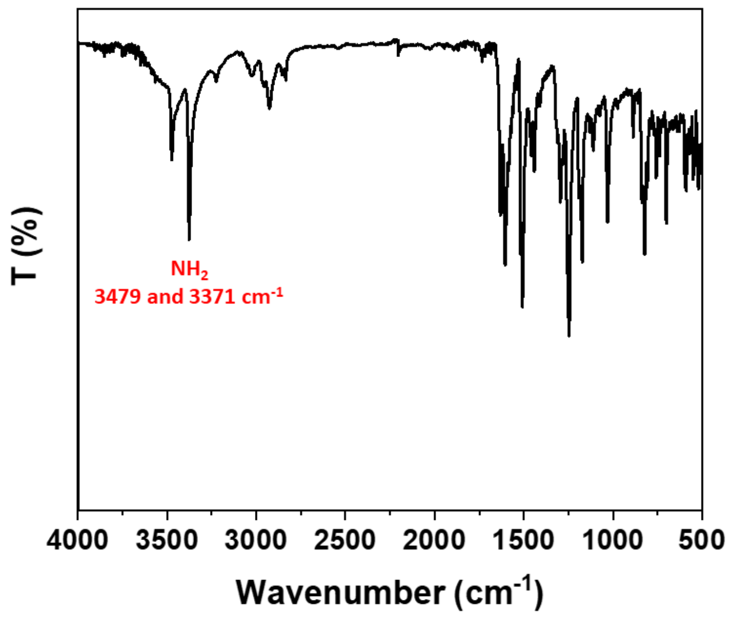

Figure S6. The FT-IR spectra of compound **4**.

6. Reference:

1- Wang, J.; Mei, J.; Yuan, W.; Lu, P.; Qin, A.; Sun, J.; Mac, Y.; Tang, B. Z. Hyperbranched polytriazoles with high molecular compressibility: aggregation-induced emission and superamplified explosive detection. *J. Mater. Chem.*, **2011**, *21*, 4056-4059.

2- Gu, X.; Yao, J.; Zhang, G.; Zhang, C.; Yan, Y.; Zhao, Y.; Zhang, D. New Electron-Donor/Acceptor-Substituted Tetraphenylethylenes: Aggregation-Induced Emission with Tunable Emission Color and Optical-Waveguide Behavior. *Chem. Asian J.*, **2013**,*8*, 2362 – 2369.

3- Inamoto, K.; Arai, Y.; Hiroya, K.; Doi, T. Palladium-catalysed direct synthesis of benzo[b]thiophenes from thioenols. *Chem. Comm.*, **2008**, 5529-5531.

4- Jana, D.; Boxi, S.; Parui, P. P.; Ghorai, B. K. Planar-Rotor Architecture Based Pyrene-Vinyl-Tetraphenylethylene Conjugated Systems: Photophysical Properties and Aggregation Behavior. *Org. Biomol. Chem.*, **2015**, *13*, 10663-10674.
